# Multi-attribute decision-making method based on complex T-spherical fuzzy frank prioritized aggregation operators

**DOI:** 10.1016/j.heliyon.2024.e25368

**Published:** 2024-02-01

**Authors:** Muhammad Rizwan khan, Kifayat Ullah, Ali Raza, Tapan Senapati, Sarbast Moslem

**Affiliations:** a Department of Mathematics, Riphah International University Lahore, Lahore, 54000, Pakistan; b School of Mathematics and Statistics, Southwest University, Chongqing, People's Republic of China; c School of Architecture Planning and Environmental Policy, University College of Dublin, D04 V1W8, Belfield, Dublin, Ireland

**Keywords:** Multi-attribute decision-making, Complex T-spherical fuzzy set, Aggregation operators, Frank t-norm and t-conorm, Decision-making

## Abstract

This article aims to introduce new aggregation operators (AOs) by assigning the positive real values known as priority degree among the strict priority levels. To Develop the complex T-spherical fuzzy (TSF) frank prioritized (CTSFFP) AOs, using the frank t-norm (FTN) and frank t-conorm (FTCN) operational laws, also explain sum, product, and power operations under complex TSF information. The TSF set framework has a superior structure for uncertain data handling than an existing intuitionistic fuzzy set (FS), Pythagorean FS (PyFS), q-rung orthopair FS (q-ROFS), picture FS (PFS), and spherical FS (SFS). Because the structure of the TSF set has the most generalized form of IFS, PyFS, q-ROFS, PFS, and SFS, it provides greater freedom to decision experts for handling information where these discussed sets fail to aggregate ambiguous details. Utilizing the idea of priority degree, proposed new AOs called CTSFFP weighted averaging (CTSFFPWA), CTSFFP ordered weighted averaging (CTSFFPOWA), CTSFFP hybrid weighted averaging (CTSFFPHWA), CTSFFP weighted geometric (CTSFFPWG), CTSFFP ordered weighted geometric (CTSFFPOWG), CTSFFP hybrid weighted geometric (CTSFFPHWG) operators. Some desirable properties of AOs, such as idempotency, monotonicity, and boundedness, are also discussed. To show the importance of proposed AOs, the real-life problem of multi-attribute decision-making (MADM) is solved with the help of developed CTSFFPWA and CTSFFPWG operators. To enhance the proposed AOs' superiority, compare the diagnosed theory with existing AOs and give conclusions.

## 1 Introduction

MADM is a technique to select the best optimal option among multiple options. According to the crisp set theory, there were only two options, "yes" or "no," in decision-making science. Due to this deficiency, many real-life problems cannot deal with crisp set theory. To cope with this problem, Zadeh [1] developed the FS with a membership degree (MD) range [0,1]. FS is used to define the confidence that lies in human opinion. Then, many mathematicians solve real-life problems using the concept of FS, such as Nasir et al. [2] solving real-world issues using decision-making sciences under the principle of FS theory and the design recognition problem solved by Chau et al. [2].

Human selection about uncertainty is not permanently unidirectional because Zadeh can only explain the MD. So, it could not give information about the result's non-membership degree (NMD). So, Atanassov [3] provides an idea for NMD. It gave the concept of an intuitionistic FS (IFS) with specific limitations. This concept helps to improve the uncertain event with the help of MD and NMD.

In many cases, the concept of IFS cannot deal with such a problem in which the sum of MD and NMD is exceeded by 1. For example, whenever the value of MD is $v_{Ѣ}=0.6$, and the value of NMD is $w_{Ѣ}=0.7$, the sum of $v_{Ѣ}$ and $w_{Ѣ}$ is 1.3, this violates FS theory. To overcome these kinds of problems, Yager [4] introduced the notion of PyFS by taking the square on $v_{Ѣ}^{2}$ and $w_{Ѣ}^{2}$ such as $0\leq v_{Ѣ}^{2}+w_{Ѣ}^{2}\leq 1$. After the passage of time the concept is also fail for bigger values of $v_{Ѣ}$ and $w_{Ѣ}$, to eliminat this issue Yager [5] proposed the notion of q-ROFS by taking the qth on $v_{Ѣ}^{q}$ and $w_{Ѣ}^{q}$ such as $0\leq v_{Ѣ}^{q}+w_{Ѣ}^{q}\leq 1$. The stucture of FS, IFS, and PyFS, and q-ROFS are unable to deal with problem in which absences degree (AD) is involved. The idea of PFS firstly introduced by the Coung [6], which he involved AD $u_{Ѣ}$, and it satisfies the condition of FS, such as $0\leq v_{Ѣ}+u_{Ѣ}+w_{Ѣ}\leq 1$. Latter on Mahmood et al. [7] extended the thought of PFS by taking the square on the MD, and NMD such as $0\leq v_{Ѣ}^{2}+u_{Ѣ}^{2}+w_{Ѣ}^{2}\leq 1$ introduced the notion SFS. After the passage of time, Mahmood et al. [7] generalized the thought of SFS by introducing the concept of TSFS.

All these above-discussed frameworks of FS cannot deal with two-dimensional and periodic information. To overcome this gap, the idea of complex FS (CFSs) with the addition of phase term in MD was developed by Ramot et al. [8]. The concept of CFS was generalized by Alkouri and Salleh [9] to give a new notion called Complex IFS (CIFS) involving MD and NMD. The extension in CIFS by taking the square on MD and NMD introduced the concept of PyFS (CPyFS) by Akram and Naz [10]. In addition, there was more advancement in the image of CPyFS by Ali and Mahmood [11] to take the qth power on the MD and NMD and introduced a new idea called complex q-RFS (Cq-ROFS). The Complex PFS (CPFS) layout was introduced by Shanthi et al. [12]. To improve this idea, Ali et al. [13] introduce the idea of Complex SFS (CSFS) and Complex TSFS (CTSFS).

In decision-making sciences, MADM is a significant topic ( Xu et al., 2024 [14]; Bouraima et al., 2024 [15]; Lo et al., 2024 [16]; Kazemi et al., 2024 [17]; Önden et al., 2024 [18]; Wang et al., 2024 [19]; Ibrahm et al., 2024 [20]; Niaz Khan et al., 2024 [21]). There were many applications of MADM in different fuzzy frameworks discussed by mathematicians. For example, the 5-G radio network selection using the MADM method with neutrosophic information given by Sharaf et al. [22] and some divergence measures for MADM in the IFS framework were introduced by Umar and Saraswat [23]. Using the trigonometric value-based AOs for MADM was diagnosed by Deveci et al. [24], and the AOs based on the interval-valued Heronian mean for the MADM problem offered by Mishra et al. [24]. The idea of complex single-valued neutrosophic sets and their procedure in MADM was diagnosed by Ali and Smaragdite [25].

Distance measuring during long route traveling problem by utilizing MADM addressed by Chen et al. [26]. The medical waste product management problem was solved by the MADM method managed by Mandal and Dutta [27]. Several geometric operators to explain the MADM issue were introduced by Wang et al. [28]. The application of MADM using Linear Diophantine FS theory was proposed by Riaz and Hashmi [29]. Recently, the application MADM for cooling system evaluation was discussed by Albahri et al. [30], and the solution to the energy consumption problem by using the interval-valued Fermenting fuzzy dominance technique provided by Jeevaraj et al. [31]. Many researchers worked a lot in decision-making environments, and the following literature is discussed in Refs. [[32], [33], [34], [35], [36]].

The MADM is a procedure for computing uncertain data to get precise outcomes. Various AOs depend on different t-norm (TN) and t-conorm (TCN), which many researchers explain. The argument on AOs depending on arithmetic operations has an extended historical group among these operators, and many AOs have been described in many fuzzy structures. Such as the concept of Dombi TN and TCN in the framework TSF by Ullah et al. [37], Sarkar and Biswas [38] introduce the concept of dual hesitant q-rung orthopair fuzzy (q-OF) Dombi TN and TCN. Wang and Liu. [39], explained Einstein's TN and TCN for weighted geometric AOs under the IFS structure. Mahnaz et al. [40] gave the concept of FTN and FTCN for TSF information and its application.

For selecting the best solar system by using the MADM method, many mathematicians explained this problem using different fuzzy structures. To rank the best working system using decision-making discussed by Akram et al. [41], introduce the performance evaluation of solar energy cells using interval-valued TSFS. Sakar and Karamian [42] introduce the model for sustainable solar PV panels using a novel integrated MADM method. Coban and Onar [43] present the analysis of solar energy generation capacity by applying hesitant fuzzy cognitive maps. Wu and Zhang [44] introduced risk assessment of offshore wave-wind solar compressed air energy storage power plants over a fuzzy all-inclusive calculation model.

FTN and FTCN are interesting fuzzy operational rules. Many authors have explored FTN and FTCN for different fuzzy frameworks. The concept of FTN and FTCN was introduced by Frank [45]. Then, the idea of straightforward operation applied to prioritized fuzzy FTN and FTCN was discussed by Rehman et al. [46]. FTN and FTCN operations for PFS were addressed by Seikh and Mandal [47], and Seikh and Mandal [48] gave the idea of FTN and FTCN for q-ROFS theory. Xu et al. [49] introduced the group decision-making technique for complex fuzzy data using frank laws. Yuqin Du [50]. developed the complex q-ROFS frank AOs and their application. In this article, we establish some AOs on Frank's operational rules. The inspiration for this article and objectives are explained as follows.

The thought of CTSFS theory, an efficient generalization of CFS, CIFS, CPyFS, Cq-ROFS, complex PFS, and CSFS, and the idea of diagnosed CTSF framework under FTN and FTCN can deal with periodic and two-dimensional data due to the additional term which is called as phase term. The Frank prioritized AOs (PAOs) as an efficient generalization of some existing AOs. That gives a helpful framework for decision-maker (DM) and provides more preciseness in outcomes than other AOs. The idea of CTSF frank PAOs offered an understanding of dealing with periodic and two-dimensional information and covered the limitations of other existing operators.

In addition, the defined AOs contain the concept of priority degree, in which evaluation experts can investigate information by prioritizing considered attributes. In the past, there had been no ideas for priority degree-based AOs for complex-valued fuzzy details. Then, we have defined the CTSFFPWA and CTSFFPWG operators using the Frank operational laws for the first time. Frank laws are the most generalized format of algebraic operations. The defined AOs are based on complex-valued information, including amplitude and phase terms. The structure of CTSFS is considered the most superior and generalized due to the involvement of AD. Because the CIFS, PyFS, and Cq-ROFS have no concept of AD and fail to handle information in which AD is involved. One of the significant features of the proposed work is that when we take the value of the phase term as zero, then the defined structure is applicable to handle all real-valued information. The developed AOs obey the fundamental axioms of AO, such as boundedness, idempotency, and monotonicity, which enhance the worth and credibility of diagnosed AOs.

This paper provides the following details: Section 2. discusses a few basic definitions. Section 3. explain some operational laws for complex TSF (CTSF) values (CTSFVs). The idea of the CTSF frank prioritized averaging (CTSFFPA) operator is discussed in Section 4. Section 5. discussed the CTSFFP weighted averaging (CTSFFPWA), CTSFFP ordered weighted averaging (CTSFPOWA), CTSFFP hybrid weighted averaging (CTSFPHWA), CTSFFP weighted geometric (CTSFFPWG), CTSFFP ordered weighted geometric (CTSFPOWG), CTSFFP hybrid weighted geometric (CTSFPHWG) operators. Section 6. describes the algorithm of MADM based on the proposed CTSFFPWA and CTSFFPWG AOs, and Section 7. provides the solution to the real-world issue of solar system selection. Section 8. provides a comparative review with current AOs. Results and discussion are addressed in Section 9. Section 10. offered conclusions.

## 2 Preliminaries

This section analyzes a few basic definitions of FS, CFS, TSFS, and CTSFS and explains their operational laws. Before discussing the fundamental definitions, construct Table 1 in which all abbreviations are defined that we needed to understand the article.

Definition 1 [1] Let Y be the non-empty set. Then a FS Ѣ can be described in Eqution (1) as follows:

(1) $$Ѣ=\left\{(y,v_{Ѣ}\left(y\right)|y\in Y\right\}$$

In this $v_{Ѣ}\left(y\right)\in \left[0,1\right]$ indicate the MD with the condition: $0\leq v_{Ѣ}\left(y\right)\leq 1$.

Definition 2 [8] Let Y be the non-empty set. Then a CFS Ѣ is described in Equation (2) as follows:

(2) $$Ѣ=\left\{(y,v_{Ѣ}\left(y\right)|y\in Y\right\}$$

In this $v_{Ѣ}\left(y\right)=v_{Ѣ}\left(y\right).e^{i2\pi \theta_{v_{Ѣ}\left(y\right)}\;}$ indicate the MD. Where $v_{Ѣ}\left(y\right)\in \left[0,1\right]$ called amplitude term and $\theta_{v_{Ѣ}\left(y\right)}\in \left[0,1\right]$ called phase term of MD.

Definition 3 [7] Let Y be the non-empty set. Then, a TSFS set Ѣ can be described in Equation (3) as follows:

(3) $$Ѣ=\left\{\left(y,v_{Ѣ}\left(y\right),u_{Ѣ}\left(y\right),w_{Ѣ}\left(y\right)\right)|y\in Y\right\}$$

In this $v_{Ѣ}\left(y\right)\in \left[0,1\right],u_{Ѣ}\left(y\right)\in \left[0,1\right]$ and $w_{Ѣ}\left(y\right)\in \left[0,1\right]$ indicate the MD, AD, and NMD, respectively, with a range of $0\leq v_{Ѣ}\left(y\right)+u_{Ѣ}\left(y\right)+w_{Ѣ}\left(y\right)\leq 1$. The condition $M_{Ѣ}\left(y\right)={\left(1-\left(v_{Ѣ}^{t}\left(y\right)+u_{Ѣ}^{t}\left(y\right)+w_{Ѣ}^{t}\left(y\right)\right)\right)}^{\frac{1}{t}}$ indicate the hesitancy degree y. Further $Ѣ=\left(v_{Ѣ},u_{Ѣ},w_{Ѣ}\right)$ is said to be TSF values (TSFVs).

Definition 4 Let Y be the non-empty set. Then, a CTSFS set Ѣ can be described in Equation (4) as follows:

(4) $$Ѣ=\left\{\left(y,v_{Ѣ}\left(y\right).\;e^{i2\pi \theta_{v_{Ѣ}\left(y\right)}},u_{Ѣ}\left(y\right).\;e^{i2\pi \varphi_{u_{Ѣ}\left(y\right)}},w_{Ѣ}\left(y\right).\;e^{i2\pi \psi_{w_{Ѣ}\left(y\right)}}\right)|y\in Y\right\}$$

Where $v_{Ѣ}\left(y\right).\;e^{i2\pi \theta_{v_{Ѣ}\left(y\right)}},u_{Ѣ}\left(y\right).\;e^{i2\pi \varphi_{u_{Ѣ}\left(y\right)}},w\left(y\right).\;e^{i2\pi \psi_{w_{Ѣ}\left(y\right)}}$ represents the MD, AD, and NMD of CTSFS with the condition $v_{Ѣ}\left(y\right)\in \left[0,1\right],u_{Ѣ}\left(y\right)\in \left[0,1\right],w\left(y\right)\in \left[0,1\right]$ known as amplitude and $\theta_{v_{Ѣ}\left(y\right)}\in \left[0,1\right],\varphi_{u_{Ѣ}\left(y\right)}\in \left[0,1\right],\psi_{w_{Ѣ}\left(y\right)}\in \left[0,1\right]$ known as phase terms of MD, AD, and NMD, respectively. Also, fulfill the following conditions such as $0\leq v_{Ѣ}\left(y\right).\;e^{i2\pi \theta_{v_{Ѣ}\left(y\right)}}+u_{Ѣ}\left(y\right).\;e^{i2\pi \varphi_{u_{Ѣ}\left(y\right)}}+w\left(y\right).\;e^{i2\pi \psi_{w_{Ѣ}\left(y\right)}}\leq 1$, $\forall \;t\epsilon Z^{+}$ and t≥1. So, we can explain the refusal degree y that is given by:

$$M_{Ѣ}\left(y\right)={\left(1-\left(v_{Ѣ}\left(y\right).\;e^{i2\pi \theta_{v_{Ѣ}\left(y\right)}}+u_{Ѣ}\left(y\right).\;e^{i2\pi \varphi_{u_{Ѣ}\left(y\right)}}+w_{Ѣ}\left(y\right).\;e^{i2\pi \psi_{w_{Ѣ}\left(y\right)}}\right)\right)}^{\frac{1}{t}}$$

For our convenience $Ѣ=\left(v_{Ѣ}\left(y\right).\;e^{i2\pi \theta_{v_{Ѣ}\left(y\right)}},u_{Ѣ}\left(y\right).\;e^{i2\pi \varphi_{u_{Ѣ}\left(y\right)}},w_{Ѣ}\left(y\right).\;e^{i2\pi \psi_{w_{Ѣ}\left(y\right)}}\right)$ is said to be CTSFV.

Definition 5 [7] Any two TSFVs ${Ѣ}_{i}=\left(v_{i},u_{i},w_{i}\right)$, i=1,2 with α>0, so

1. ${Ѣ}_{1}⊕{Ѣ}_{2}=\left({\left(v_{1}^{t}+v_{2}^{t}-v_{1}^{t}v_{2}^{t}\right)}^{\frac{1}{t}},\left(u_{1}u_{2}\right),\left(w_{1}w_{2}\right)\right)$.
2. ${Ѣ}_{1}⊗{Ѣ}_{2}=\left(\left(v_{1}v_{2}\right),{\left(u_{1}^{t}+u_{2}^{t}-u_{1}^{t}u_{2}^{t}\right)}^{\frac{1}{t}},{\left(w_{1}^{t}+w_{2}^{t}-w_{1}^{t}w_{2}^{t}\right)}^{\frac{1}{t}}\right)$.
3. $\alpha^{{Ѣ}_{i}}=\left({\left(1-{\left(1-v_{i}^{t}\right)}^{\alpha }\right)}^{\frac{1}{t}},u_{i}^{t},w_{j}^{t}\right)$.
4. ${Ѣ}_{i}^{\alpha }=\left(v_{i}^{t},{\left(1-\;{\left(1-u_{i}^{t}\right)}^{\alpha }\right)}^{\frac{1}{t}},{\left(1-{\left(1-w_{i}^{t}\right)}^{\alpha }\right)}^{\frac{1}{t}}\right)$.

Definition 6 [51,52] For CTSFVs ${Ѣ}_{i}=\left(v_{Ѣ}\left(y\right).\;e^{i2\pi \theta_{v_{Ѣ}\left(y\right)}},u_{Ѣ}\left(y\right).\;e^{i2\pi \varphi_{u_{Ѣ}\left(y\right)}},w_{Ѣ}\left(y\right).\;e^{i2\pi \psi_{w_{Ѣ}\left(y\right)}}\right)$, the score values (SV) in defined in Equation (5) and accuracy value (AV) in defined in Equation (6) as follows:

(5) $$S\left({Ѣ}_{i}\right)=\frac{2+\left[\left(v_{Ѣ}\left(y\right)-u_{Ѣ}\left(y\right)-w\left(y\right)\right)+\left(\theta_{v_{Ѣ}\left(y\right)}-\varphi_{u_{Ѣ}\left(y\right)}-\psi_{w_{Ѣ}\left(y\right)}\right)\right]}{4}$$

(6) $$H\left({Ѣ}_{i}\right)=\frac{2+\left[\left(v_{Ѣ}\left(y\right)+u_{Ѣ}\left(y\right)+w\left(y\right)\right)+\left(\theta_{v_{Ѣ}\left(y\right)}+\varphi_{u_{Ѣ}\left(y\right)}+\psi_{w_{Ѣ}\left(y\right)}\right)\right]}{4}$$

Definition 7 [7] In this $S\left({Ѣ}_{i}\right)=\left[-1,1\right]$, $H\left({Ѣ}_{i}\right)=\left[0,1\right]$. The connection between any two CTSFVs ${Ѣ}_{i}$ and ${Ѣ}_{i}^{`}$ can be described as;

1. Condition $S\left({Ѣ}_{i}\right)>S\left({Ѣ}_{i}^{`}\right)$ then ${Ѣ}_{i}>{Ѣ}_{i}^{`}$.
2. Condition $S\left({Ѣ}_{i}\right)=S\left({Ѣ}_{i}^{`}\right)$, then
a. Condition $H\left({Ѣ}_{i}\right)>H\left({Ѣ}_{i}^{`}\right)$ then ${Ѣ}_{i}>{Ѣ}_{i}^{`}$.
b. Condition $H\left({Ѣ}_{i}\right)=H\left({Ѣ}_{i}^{`}\right)$ then ${Ѣ}_{i}={Ѣ}_{i}^{`}$.

**Table 1:** Explanation of abbreviations.

FS	Fuzzy set	IFS	Intuitionistic fuzzy set
CFS	Complex fuzzy set	CIFS	Complex Intuitionistic fuzzy set
TSFS	T-spherical fuzzy set	PyFS	Pythagorean fuzzy set
CTSFS	Complex T-spherical fuzzy set	q-ROFS	q-rung orthopair fuzzy set
SFS	Spherical fuzzy set	AOs	Aggregation operators
FTNM	frank t-norm	AD	Absence degree
FTCNM	frank t-conorm	NMD	non-membership degree
MD	Membership degree		
CTSFFP	Complex T-spherical fuzzy frank prioritized	CTSFFPWG	Complex T-spherical fuzzy frank prioritized weighted geometric
CTSFFPWA	Complex T-spherical fuzzy frank prioritized weighted averaging	CTSFFPOWG	Complex T-spherical fuzzy frank prioritized ordered weighted geometric
CTSFFPOWA	Complex T-spherical fuzzy frank prioritized ordered weighted averaging	CTSFFPHWG	Complex T-spherical fuzzy frank prioritized hybrid weighted geometric
CTSFFPHWA	Complex T-spherical fuzzy frank prioritized hybrid weighted averaging	MADM	Multi-attribute decision-making
CSFS	Complex spherical fuzzy set	Cq-ROFS	Complex q-Rung orthopair fuzzy set
CPFS	Complex Picture fuzzy set	q-ROFS	q-Rung orthopair fuzzy set
CTSFDWA	Complex T-spherical fuzzy Dombi weighted averaging	CTSFDWG	Complex T-spherical fuzzy Dombi weighted geometric
WA	Weighted Averaging	WG	Weighted geometric
CTSFHWA	Complex T-spherical fuzzy Hamacher weighted averaging	CTSFHWG	Complex T-spherical fuzzy Hamacher weighted geometric
BSFS	Bipolar soft fuzzy set	LPyFS	Linguistic Pythagorean fuzzy set
MSM	Maclaurin symmetric mean	q-ROF	q-rung orthopair
q-ROFAAWG	q-rung orthopair Aczel Alsina weighted Averaging weighted geometric	q-ROFAAWA	q-rung orthopair Aczel Alsina weighted Averaging weighted averaging
DM	Decision-maker	PAOs	Prioritized aggregation operators

## 3 Frank aggregation operators for CTSFV information

This section discussed the frank TN and TCN based on the CTSFV information. We discuss two frank TN and TCN cases: frank products and frank sum.

Definition 8 [45] The function from ${\left[0,1\right]}^{2}\to \left[0,1\right]$ are frank TN discussed in Equation (7) and TCN explained in Equation (8) as follows:

(7) $$E_{o}\left(\rho ,\mu \right)=\log_{o}\left(1+\frac{\left(o^{\rho }-1\right)\left(o^{\mu }-1\right)}{o-1}\right),\rho ,\sigma \in {\left[0,1\right]}^{2},o>1$$

(8) $$F_{o}\left(\rho ,\mu \right)=\log_{o}\left(1+\frac{\left(o^{1-\rho }-1\right)\left(o^{1-\mu }-1\right)}{o-1}\right),\rho ,\sigma \in {\left[0,1\right]}^{2},o>1$$

Using the TCN and TN operations in the next step, we define the generalized form of union and intersection of two CTSFVs as follows.

Definition 9 Assume that $R_{i}=\left\{a,v_{R_{i}}\left(y\right).\;e^{i2\pi {\theta_{v}}_{R_{i}\left(y\right)}},u_{R_{i}}\left(y\right).\;e^{i2\pi {\varphi_{u}}_{R_{i}\left(y\right)}},w_{R_{i}}\left(y\right).\;e^{i2\pi {\psi_{w}}_{R_{i}\left(y\right)}},a\in A\right\}$ for i=1,2,3,… be CTSFVs, So the union and intersection of the CTSF frank value (CTSFFVs) are explained in Equation (9) and in Equation (10) respectively:

(9) $$U_{1}\cup U_{2}=\left\{\begin{array}{c} a,F\left(v_{u_{1}}\left(a\right),e^{i2\pi \theta_{v_{u_{1}}}\left(a\right)},v_{u_{2}}\left(a\right),e^{i2\pi \theta_{v_{u_{2}}}\left(a\right)}\right)\\ \;E\left(u_{u_{1}}\left(a\right),e^{i2\pi \varphi_{u_{u_{1}}}\left(a\right)},u_{u_{2}}\left(a\right),e^{i2\pi \varphi_{u_{u_{2}}}\left(a\right)}\right),\\ \;E\left(w_{u_{1}}\left(a\right),e^{i2\pi \psi_{w_{u_{1}}}\left(a\right)},u_{u_{2}}\left(a\right),e^{i2\pi \psi_{w_{u_{2}}}\left(a\right)}\right) \end{array}\;a\in A\right\}$$

(10) $$U_{1}\cap U_{2}=\left\{\begin{array}{c} a,E\left(v_{u_{1}}\left(a\right),e^{i2\pi \theta_{v_{u_{1}}}\left(a\right)},v_{u_{2}}\left(a\right),e^{i2\pi \theta_{v_{u_{2}}}\left(a\right)}\right)\\ \;F\left(u_{u_{1}}\left(a\right),e^{i2\pi \varphi_{u_{u_{1}}}\left(a\right)},u_{u_{2}}\left(a\right),e^{i2\pi \varphi_{u_{u_{2}}}\left(a\right)}\right),\\ \;F\left(w_{u_{1}}\left(a\right),e^{i2\pi \psi_{w_{u_{1}}}\left(a\right)},u_{u_{2}}\left(a\right),e^{i2\pi \psi_{w_{u_{2}}}\left(a\right)}\right) \end{array}\;a\in A\right\}$$

Definition 10 Suppose $T_{1}=\left(v_{1}.\;e^{i2\pi \theta_{v_{1}}},u_{1}.\;e^{i2\pi \varphi_{u_{1}}},w_{1}.\;e^{i2\pi \psi_{w_{1}}}\right)$ and $T_{2}=\left(v_{2}.\;e^{i2\pi \theta_{v_{2}}},u_{2}.\;e^{i2\pi \varphi_{u_{2}}},w_{2}.\;e^{i2\pi \psi_{w_{2}}}\right)$ be two CTSFFVs. The sum and product for $T_{1}$ and $T_{2}$ can be defined in Equation (11) and in Equation (12) as follows:

(11) $$T_{1}{⊕}_{E,F}T_{2}=\left\{F\left(v_{1}.e^{i2\pi \theta_{v_{1}}},v_{2}.e^{i2\pi \theta_{v_{2}}}\right),E\left(u_{1}.e^{i2\pi \varphi_{u_{1}}},u_{2}.e^{i2\pi \varphi_{u_{2}}}\right),E\left(w_{1}.e^{i2\pi \psi_{w_{1}}},w_{2}.e^{i2\pi \psi_{w_{2}}}\right)\right\}$$

(12) $$T_{1}{⊗}_{E,F}T_{2}=\left\{E\left(v_{1}.e^{i2\pi \theta_{v_{1}}},v_{2}.e^{i2\pi \theta_{v_{2}}}\right),F\left(u_{1}.e^{i2\pi \varphi_{u_{1}}},u_{2}.e^{i2\pi \varphi_{u_{2}}}\right),F\left(w_{1}.e^{i2\pi \psi_{w_{1}}},w_{2}.e^{i2\pi \psi_{w_{2}}}\right)\right\}$$

Definition 11 Assume, $A_{f}$ is a TN and $Z_{f}$ is a TCN, and let $A_{f}$ be a frank product, and $Z_{f}$ be the frank sum of the CTSFVs. We can suppose two numbers. $q_{1}=\left({v_{1}}_{Ѣ}.\;e^{i2\pi \theta_{{v_{1}}_{Ѣ}}},{u_{1}}_{Ѣ}.\;e^{i2\pi \varphi_{{u_{1}}_{Ѣ}}},{w_{1}}_{Ѣ}.\;e^{i2\pi \psi_{{w_{1}}_{Ѣ}}}\right)$ and $q_{2}=\left({v_{2}}_{Ѣ}.\;e^{i2\pi \theta_{{v_{2}}_{Ѣ}}},{u_{2}}_{Ѣ}.\;e^{i2\pi \varphi_{{u_{2}}_{Ѣ}}},{w_{2}}_{Ѣ}.\;e^{i2\pi \psi_{{w_{2}}_{Ѣ}}}\right)$. So, $q_{1}{⊕}_{f}q_{2}$ and $q_{1}{⊗}_{f}q_{2}$ can represent the frank sum, product, scalar multiplication and power operations are discussed in Equation (13), (14), (15), (16) as follows:

(13) $$q_{1}{⊕}_{f}q_{2}=\left(\begin{array}{c} \sqrt[{t}]{1-\log_{o}\left(1+\frac{\left(o^{1-{\left({v_{1}}_{Ѣ}\right)}^{t}}-1\right)\left(o^{1-{\left({v_{2}}_{Ѣ}\right)}^{t}}-1\right)}{o-1}\right)},\\ e^{i2\pi \sqrt[{t}]{1-\log_{o}\left(1+\frac{\left(o^{1-{\left(\theta_{{v_{1}}_{Ѣ}}\right)}^{t}}-1\right)\left(o^{1-{\left(\theta_{{v_{2}}_{Ѣ}}\right)}^{t}}-1\right)}{o-1}\right)}}\\ \log_{o}\begin{array}{c} \left(1+\frac{\left(o^{{\left({u_{1}}_{Ѣ}\right)}^{t}}-1\right)\left(o^{{\left({u_{2}}_{Ѣ}\right)}^{t}}-1\right)}{o-1}\right),\\ e^{i2\pi \;\log_{o}\left(1+\frac{\left(o^{{\left(\varphi_{{u_{1}}_{Ѣ}}\right)}^{t}}-1\right)\left(o^{{\left(\varphi_{{u_{2}}_{Ѣ}}\right)}^{t}}-1\right)}{o-1}\right)} \end{array}\\ \log_{o}\begin{array}{c} \left(1+\frac{\left(o^{{\left({w_{1}}_{Ѣ}\right)}^{t}}-1\right)\left(o^{{\left({w_{2}}_{Ѣ}\right)}^{t}}-1\right)}{o-1}\right),\\ e^{i2\pi \;\log_{o}\left(1+\frac{\left(o^{{\left(\psi_{{w_{1}}_{Ѣ}}\right)}^{t}}-1\right)\left(o^{{\left(\psi_{{w_{2}}_{Ѣ}}\right)}^{t}}-1\right)}{o-1}\right)} \end{array} \end{array}\right)o>1$$

(14) $$q_{1}{⊗}_{f}q_{2}=\left(\begin{array}{c} \log_{o}\begin{array}{c} \left(1+\frac{\left(o^{{\left({v_{1}}_{Ѣ}\right)}^{t}}-1\right)\left(o^{{\left({v_{2}}_{Ѣ}\right)}^{t}}-1\right)}{o-1}\right),\\ e^{i2\pi \;\log_{o}\left(1+\frac{\left(o^{{\left(\theta_{{v_{1}}_{Ѣ}}\right)}^{t}}-1\right)\left(o^{{\left(\theta_{{v_{2}}_{Ѣ}}\right)}^{t}}-1\right)}{o-1}\right)} \end{array}\\ \sqrt[{t}]{1-\log_{o}\left(1+\frac{\left(o^{1-{\left({u_{1}}_{Ѣ}\right)}^{t}}-1\right)\left(o^{1-{\left({u_{2}}_{Ѣ}\right)}^{t}}-1\right)}{o-1}\right)},\\ e^{i2\pi \sqrt[{t}]{1-\log_{o}\left(1+\frac{\left(o^{1-{\left(\varphi_{{u_{1}}_{Ѣ}}\right)}^{t}}-1\right)\left(o^{1-{\left(\varphi_{{u_{2}}_{Ѣ}}\right)}^{t}}-1\right)}{o-1}\right)}}\\ \sqrt[{t}]{1-\log_{o}\left(1+\frac{\left(o^{1-{\left({w_{1}}_{Ѣ}\right)}^{t}}-1\right)\left(o^{1-{\left({w_{2}}_{Ѣ}\right)}^{t}}-1\right)}{o-1}\right)},\\ e^{i2\pi \sqrt[{t}]{1-\log_{o}\left(1+\frac{\left(o^{1-{\left(\psi_{{w_{1}}_{Ѣ}}\right)}^{t}}-1\right)\left(o^{1-{\left(\psi_{{w_{2}}_{Ѣ}}\right)}^{t}}-1\right)}{o-1}\right)}} \end{array}\right)o>1$$

(15) $$\alpha .q_{i}=\left(\begin{array}{c} {\left(1-{\left(1-\left(\sqrt[{t}]{1-\log_{o}\left(1+\frac{\left(o^{1-{\left(v_{i}\right)}^{t}}-1\right)}{o-1}\right)}\right)\right)}^{\alpha }\right)}^{\frac{1}{t}},\\ e^{2\pi i{\left(1-{\left(1-\left(\sqrt[{t}]{1-\log_{o}\left(1+\frac{\left(o^{1-{\left({\theta_{v}}_{i}\right)}^{t}}-1\right)}{o-1}\right)}\right)\right)}^{\alpha }\right)}^{\frac{1}{t}}},\\ \log_{o}\left(1+\frac{\left(o^{{\left(u_{i}\right)}^{t}}-1\right)}{o-1}\right),e^{i2\pi \;\log_{o}\left(1+\frac{\left(o^{{\left({\varphi_{u}}_{i}\right)}^{t}}-1\right)}{o-1}\right)},\\ \log_{o}\left(1+\frac{\left(o^{{\left(w_{i}\right)}^{t}}-1\right)}{o-1}\right),e^{i2\pi \;\log_{o}\left(1+\frac{\left(o^{{\left({\psi_{w}}_{i}\right)}^{t}}-1\right)}{o-1}\right)} \end{array}\right)$$

(16) $$q_{i}^{\alpha }=\left(\begin{array}{c} \log_{o}\left(1+\frac{\left(o^{{\left(v_{i}\right)}^{t}}-1\right)}{o-1}\right),e^{i2\pi \;\log_{o}\left(1+\frac{\left(o^{{\left(\theta_{v}\right)}^{t}}-1\right)}{o-1}\right)},\\ \left(\begin{array}{c} {\left(1-{\left(1-\sqrt[{t}]{1-\log_{o}\left(1+\frac{\left(o^{1-{\left(u_{i}\right)}^{t}}-1\right)}{o-1}\right)}\right)}^{\alpha }\right)}^{\frac{1}{t}},\\ e^{2\pi i{\left(1-{\left(1-\sqrt[{t}]{1-\log_{o}\left(1+\frac{\left(o^{1-{\left({\varphi_{u}}_{i}\right)}^{t}}-1\right)}{o-1}\right)}\right)}^{\alpha }\right)}^{\frac{1}{t}}} \end{array}\right),\\ \left(\begin{array}{c} {\left(1-{\left(1-\sqrt[{t}]{1-\log_{o}\left(1+\frac{\left(o^{1-{\left(w_{i}\right)}^{t}}-1\right)}{o-1}\right)}\right)}^{\alpha }\right)}^{\frac{1}{t}},\\ e^{2\pi i{\left(1-{\left(1-\sqrt[{t}]{1-\log_{o}\left(1+\frac{\left(o^{1-{\left({\psi_{w}}_{i}\right)}^{t}}-1\right)}{o-1}\right)}\right)}^{\alpha }\right)}^{\frac{1}{t}}} \end{array}\right) \end{array}\right)$$

## 4 Proposed CTSFFPA operators

This part discusses basic definitions of the prioritized AOs in the CTSF surrounding based on FTN and FTC.

### 4.1 CTSF prioritized operator

Suppose ${Ѣ}_{i}=\left(v_{i},u_{i},w_{i}\right)$ is the collection of the CTSFFPA have accurate prioritization follow ${Ѣ}_{1}{>}_{m_{1}}{Ѣ}_{2}{>}_{m_{2}}\ldots {>}_{m_{n-1}}{Ѣ}_{i}$ ${Ѣ}_{i}$ in this ${Ѣ}_{i}{>}_{m_{i}}{Ѣ}_{i+1}$, this expression shows that CTSFVs ${Ѣ}_{i}$ having a $m_{i}$ huge priority than the ${Ѣ}_{i+1}$. The vector of priority degree vector $m_{i}=\left(m_{1},m_{2},\ldots ,m_{n}\right)$ follows $0\leq m_{ii}<\infty$. We can explain these values such as CTSFFPA has priority accurate with priority degree as ${ʒ}_{m}$.

Definition 12 Consider that the CTSFFPA_m_ operator is a function $CTSFFPA:{ʒ}_{m}^{z}⟶{ʒ}_{m}$ explained as follows in Equation (17):

(17) $$CTSFFPA\left({Ѣ}_{1},{Ѣ}_{2},\ldots ,{Ѣ}_{z}\right)=y_{1}^{m}{Ѣ}_{1}⊕y_{2}^{m}{Ѣ}_{2}⊕\ldots y_{z}^{m}{Ѣ}_{z}$$

In this section, $R_{i}^{m}=\frac{T_{i}^{m}}{\sum_{i=1}^{z}T_{i}^{m}},T_{i}^{m}=\prod_{p=1}^{i-1}S{\left({Ѣ}_{p}\right)}^{m}$ and $T_{1}=1$. So, the CTSFFPA operator is also called the CTSFFPA operator priority degree.

Theorem 1 Consider ${Ѣ}_{i}=\left(v_{i}.\;e^{i2\pi \theta_{v_{i}}},u_{i}.\;e^{i2\pi \varphi_{u_{i}}},w_{i}.\;e^{i2\pi \psi_{w_{i}}}\right)$ be CTSFFVs, then using CTSFFPA_m_ AOs, the resulting values are also CTSFFPVs.

$$CTSFFPA\left({Ѣ}_{1},{Ѣ}_{2},\ldots ,{Ѣ}_{z}\right)=\left(\begin{array}{c} \sqrt[{t}]{1-\log_{o}\left(1+\prod_{i=1}^{z}{\left(\left(o^{1-{\left(v_{i}\right)}^{t}}-1\right)\right)}^{{\left(R_{i}\right)}^{m}}\right)},e^{i2\pi \sqrt[{t}]{1-\log_{o}\left(1+\prod_{i=1}^{z}{\left(\left(o^{1-{\left(\theta_{v_{i}}\right)}^{t}}-1\right)\right)}^{{\left(R_{i}\right)}^{m}}\right)},}\\ \log_{o}\left(1+\prod_{i=1}^{z}{\left(\left(o^{{\left(u_{i}\right)}^{t}}-1\right)\right)}^{{\left(R_{i}\right)}^{m}}\right),e^{i2\pi \;\log_{o}\left(1+\prod_{i=1}^{z}{\left(\left(o^{{\left(\varphi_{u_{i}}\right)}^{t}}-1\right)\right)}^{{\left(R_{i}\right)}^{m}}\right)}\\ \log_{o}\left(1+\prod_{i=1}^{z}{\left(\left(o^{{\left(w_{i}\right)}^{t}}-1\right)\right)}^{{\left(R_{i}\right)}^{m}}\right),e^{i2\pi \;\log_{o}\left(1+\prod_{i=1}^{z}{\left(\left(o^{{\left(\psi_{w_{i}}\right)}^{t}}-1\right)\right)}^{{\left(R_{i}\right)}^{m}}\right)} \end{array}\right)$$

**Proof:** The Theorem can be proved by using Definition 11. Theorem 1. satisfies idempotency, boundedness, and monotonic property under CTSFFVs information.

**Example 1.** Assume the four sets of the CTSFFVs $\rho_{1},\rho_{2},\rho_{3}\;\text{and}\;\rho_{4}$. Then, consider $\rho_{1}=\left(\left(0.69,0.5\right),\left(0.65,0.85\right),\left(0.36,0.58\right)\right)$, $\rho_{2}=\left(\left(0.52,0.56\right),\left(0.56,0.86\right),\left(0.93,0.52\right)\right)$, $\rho_{3}=\left(\left(0.25,0.56\right),\left(0.58,0.38\right),\left(0.86,0.74\right)\right)$ and $\rho_{4}=\left(\left(0.58,0.69\right),\left(0.56,0.13\right),\left(0.16,0.82\right)\right)$. First of all, using Definition 12. Find the SV $S\left(\rho_{1}\right)=0.3647$, $S\left(\rho_{2}^{`}\right)=0.1762$, $S\left(\rho_{3}\right)=0.2804$ and $S\left(\rho_{4}\right)=0.4471$. Then, we find the value ${Ѣ}_{1}\left(i=1,2,3,4\right)$ for unlike four priority vectors $m_{i}=\left(m_{1},m_{2},m_{3}\right)$ with condition $m_{2}$, $m_{3}$ will be the same, but $m_{1}$ can be varied. Then,

Condition 1 $m_{1}=\left(1,1,1\right)$, then we calculate $T_{2}^{m}={\left(S\left(\rho_{1}\right)\right)}^{m_{1}}=0.3647$, $T_{3}^{m}={\left(S\left(\rho_{1}\right)\right)}^{m_{1}}\times {\left(S\left(\rho_{2}\right)\right)}^{m_{2}}=0.0643$$T_{4}^{m}={\left(S\left(\rho_{1}\right)\right)}^{m_{1}}\times {\left(S\left(\rho_{2}\right)\right)}^{m_{2}}\times {\left(S\left(\rho_{3}\right)\right)}^{m_{3}}=0.0180$ and $T_{1}^{m}=1$. Then $\sum_{p=1}^{4}T_{p}=1+0.3647+0.0643+0.0180=1.4470$. Thus using $R_{1}^{m}=0.6911$, $R_{2}^{m}=0.2520$, $R_{3}^{m}=0.0444$ and $R_{4}^{m}=0.0125\text{,}$ we use the following expression,

$$CTSFFPA\left({Ѣ}_{1},{Ѣ}_{2},\ldots ,{Ѣ}_{z}\right)=\left(\begin{array}{c} \sqrt[{t}]{1-\log_{o}\left(1+\prod_{i=1}^{z}{\left(\left(o^{1-{\left(v_{i}\right)}^{t}}-1\right)\right)}^{{\left(R_{i}\right)}^{m}}\right)},e^{i2\pi \sqrt[{t}]{1-\log_{o}\left(1+\prod_{i=1}^{z}{\left(\left(o^{1-{\left(\theta_{v_{i}}\right)}^{t}}-1\right)\right)}^{{\left(R_{i}\right)}^{m}}\right)},}\\ \log_{o}\left(1+\prod_{i=1}^{z}{\left(\left(o^{{\left(u_{i}\right)}^{t}}-1\right)\right)}^{{\left(R_{i}\right)}^{m}}\right),e^{i2\pi \;\log_{o}\left(1+\prod_{i=1}^{z}{\left(\left(o^{{\left(\varphi_{u_{i}}\right)}^{t}}-1\right)\right)}^{{\left(R_{i}\right)}^{m}}\right)}\\ \log_{o}\left(1+\prod_{i=1}^{z}{\left(\left(o^{{\left(w_{i}\right)}^{t}}-1\right)\right)}^{{\left(R_{i}\right)}^{m}}\right),e^{i2\pi \;\log_{o}\left(1+\prod_{i=1}^{z}{\left(\left(o^{{\left(\psi_{w_{i}}\right)}^{t}}-1\right)\right)}^{{\left(R_{i}\right)}^{m}}\right)} \end{array}\right)$$

$$=\sqrt[{t}]{1-\log_{o}\left(1+\prod_{i=1}^{z}{\left(\left(o^{1-{\left(v_{i}\right)}^{t}}-1\right)\right)}^{{\left(R_{i}\right)}^{m}}\right)},e^{i2\pi \sqrt[{t}]{1-\log_{o}\left(1+\prod_{i=1}^{z}{\left(\left(o^{1-{\left(\theta_{v_{i}}\right)}^{t}}-1\right)\right)}^{{\left(R_{i}\right)}^{m}}\right)},}$$

$$=\sqrt[{4}]{1-\log_{2}\left(1+\left(\begin{array}{c} {\left(2^{1-\left(0.69\right)}-1\right)}^{0.611}\times \\ {\left(2^{1-\left(0.52\right)}\right)}^{0.2520}\\ \times {\left(2^{1-0.25}\right)}^{0.0444}\\ \times {\left(2^{1-0.58}\right)}^{0.0125} \end{array}\right)\right)\;},e^{i2\pi \sqrt[{4}]{1-\log_{2}\left(1+\left(\begin{array}{c} {\left(2^{1-\left(0.69\right)}-1\right)}^{0.611}\\ \times {\left(2^{1-\left(0.52\right)}\right)}^{0.2520}\\ \times {\left(2^{1-0.25}\right)}^{0.0444}\\ \times {\left(2^{1-0.58}\right)}^{0.0125} \end{array}\right)\right)}}$$

=(0.6686,0.5127)

Same as we can calculate for $\log_{o}\left(1+\prod_{i=1}^{z}{\left(\left(o^{{\left(u_{i}\right)}^{t}}-1\right)\right)}^{{\left(R_{i}\right)}^{m}}\right),e^{i2\pi \;\log_{o}\left(1+\prod_{i=1}^{z}{\left(\left(o^{{\left(\varphi_{u_{i}}\right)}^{t}}-1\right)\right)}^{{\left(R_{i}\right)}^{m}}\right)}=\left(0.1613,0.4912\right)$ and $\log_{o}\left(1+\prod_{i=1}^{z}{\left(\left(o^{{\left(w_{i}\right)}^{t}}-1\right)\right)}^{{\left(R_{i}\right)}^{m}}\right),e^{i2\pi \;\log_{o}\left(1+\prod_{i=1}^{z}{\left(\left(o^{{\left(\psi_{w_{i}}\right)}^{t}}-1\right)\right)}^{{\left(R_{i}\right)}^{m}}\right)}=\left(0.0336,0.1083\right)$.

$$CTSFFPA\left({Ѣ}_{1},{Ѣ}_{2},\ldots ,{Ѣ}_{z}\right)=\left(\left(0.6686,0.5127\right),\left(0.1613,0.4912\right),\left(0.0336,0.1083\right)\right)$$

Condition 2 $m_{1}=\left(4,1,1\right)$, then follow the same step as Condition 1. Then find

$$CTSFFPA\left({Ѣ}_{1},{Ѣ}_{2},\ldots ,{Ѣ}_{z}\right)=\left(\left(0.6881,0.5113\right),\left(0.1767,0.5224\right),\left(0.0180,0.1123\right)\right)$$

Condition 3 $m_{1}=\left(8,1,1\right)$.

$$CTSFFPA\left({Ѣ}_{1},{Ѣ}_{2},\ldots ,{Ѣ}_{z}\right)=\left(\left(0.69,0.5\right),\left(0.1785,0.5220\right),\left(0.0168,0.1132\right)\right)$$

Condition 4 $m_{1}=\left(12,1,1\right)$.

$$CTSFFPA\left({Ѣ}_{1},{Ѣ}_{2},\ldots ,{Ѣ}_{z}\right)=\left(\left(0.69,0.5\right),\left(0.1785,0.5220\right),\left(0.0168,0.1132\right)\right)$$

In all conditions, we can find the value of CTSFFPWA on the base of the degree vector.

## 5 CTSFFP aggregation operator

This portion discusses the explanation of weighted averaging and geometric AOs and the basic concept based on the CTSFFPA operator.

### 5.1 CTSFFP weighted averaging operators

This section consists of CTSFFPWA AOs and the basic concept of CTSFFPOWA and CTSFFPHWA AOs.

Definition 13 Suppose $q_{i}=\left(v_{i}.\;e^{i2\pi \theta_{v_{i}}},u_{i}.\;e^{i2\pi \varphi_{u_{i}}},w_{i}.\;e^{i2\pi \psi_{w_{i}}}\right)$ be some CTSFVs and $R_{i}={\left(r_{1},r_{2},\ldots ,r_{n}\right)}^{t}$ c be the weight vector (WV) of the $q_{i}$ condition on weight is $R_{i}\geq 0$ and $\sum_{i}^{n}R_{i}=1$. Then, the CTSFFPWA operator is $l^{*n}⟶l^{*}$, defined as:

$$\text{CTSFFPWA}\left(q_{1},q_{2},\ldots ,q_{n}\right)={⊕}_{i=1}^{n}q_{i}R_{i}=R_{1}q_{1}⊕R_{2}q_{2}⊕\ldots ⊕R_{n}q_{n}$$

We utilize the frank operation on the CTSFFPA operator in the following theorem.

Theorem 2 Assume that $q_{i}=\left(v_{i}.\;e^{i2\pi \theta_{v_{i}}},u_{i}.\;e^{i2\pi \varphi_{u_{i}}},w_{i}.\;e^{i2\pi \psi_{w_{i}}}\right)$ be CTSFVs. By applying the operation of CTSFFPWA to evaluate the value of $q_{i}`s$. And aggregation results are also CTSFVs and presented as in Equation (18):

(18) $$\text{CTSFFPWA}\left(q_{1},q_{2},\ldots ,q_{n}\right)=\left(\begin{array}{c} \sqrt[{t}]{1-\log_{o}\left(1+\prod_{i=1}^{n}{\left(\left(o^{1-{\left(v_{i}\right)}^{t}}-1\right)\right)}^{R_{i}}\right)},e^{i2\pi \sqrt[{t}]{1-\log_{o}\left(1+\prod_{i=1}^{n}{\left(\left(o^{1-{\left(\theta_{v_{i}}\right)}^{t}}-1\right)\right)}^{R_{i}}\right)},}\\ \log_{o}\left(1+\prod_{i=1}^{n}{\left(\left(o^{{\left(u_{i}\right)}^{t}}-1\right)\right)}^{R_{i}}\right),e^{i2\pi \;\log_{o}\left(1+\prod_{i=1}^{n}{\left(\left(o^{{\left(\varphi_{u_{i}}\right)}^{t}}-1\right)\right)}^{R_{i}}\right)}\\ \log_{o}\left(1+\prod_{i=1}^{n}{\left(\left(o^{{\left(w_{i}\right)}^{t}}-1\right)\right)}^{R_{i}}\right),e^{i2\pi \;\log_{o}\left(1+\prod_{i=1}^{n}{\left(\left(o^{{\left(\psi_{w_{i}}\right)}^{t}}-1\right)\right)}^{R_{i}}\right)} \end{array}\right)$$

*The proof is provided in the appendix*.

*To investigate*, *the proposed AOs satisfy the following axioms*: *idempotency*, *boundedness*, *and monotonicity*, *along with detailed proofs*, *which enhance the worth of suggested operators*.

Theorem 3 (Idempotency) Consider $q_{i}=\left(v_{i}.\;e^{i2\pi \theta_{v_{i}}},u_{i}.\;e^{i2\pi \varphi_{u_{i}}},w_{i}.\;e^{i2\pi \psi_{w_{i}}}\right)=\left(v.e^{2\pi i\theta_{v}},u.e^{2\pi i\varphi_{u}},w.e^{2\pi i\psi_{w}}\right)=q$.

*The proof is provided in the appendix*.

Theorem 4 (*Boundedness*) *Consider* $q_{i}=\left(v_{i}.\;e^{i2\pi \theta_{v_{i}}},u_{i}.\;e^{i2\pi \varphi_{u_{i}}},w_{i}.\;e^{i2\pi \psi_{w_{i}}}\right)$ *be an AOs for CTSFFVs*. *Suppose* $q_{i}^{-}=\min\left(q_{1},q_{2},\ldots ,q_{n}\right)$ *and* $q_{i}^{+}=\max\left(q_{1},q_{2},\ldots ,q_{n}\right)$. *So*

$$q_{i}^{-}\leq CTSFFPWA\left(q_{1},q_{2},\ldots ,q_{n}\right)\leq q_{i}^{+}$$

*The proof is****provided****in the appendix*.

Theorem 5 (Monotonicity) Consider $q_{i}=\left(v_{i}.\;e^{i2\pi \theta_{v_{i}}},u_{i}.\;e^{i2\pi \varphi_{u_{i}}},w_{i}.\;e^{i2\pi \psi_{w_{i}}}\right)$ and $\overset{´}{q_{i}}=\left(\overset{´}{v_{i}}.\;e^{i2\pi \overset{´}{\theta_{v_{i}}}},\overset{´}{u_{i}}.\;e^{i2\pi \overset{´}{\varphi_{u_{i}}}},\overset{´}{w_{i}}.\;e^{i2\pi \overset{´}{\psi_{w_{i}}}}\right)$ i=(1,2,…,n) be two CTSFFVs, such as $q_{i}\leq \overset{´}{q_{i}}$ where $v_{i}\leq \overset{´}{v_{i}},\theta_{v_{i}}\leq \overset{´}{\theta_{v_{i}}}$, $u_{i}\geq \overset{´}{u_{i}},\varphi_{u_{i}}\geq \overset{´}{\varphi_{u_{i}}}$ and $w_{i}\geq \overset{´}{w_{i}},\psi_{w_{i}}\geq \overset{´}{\psi_{w_{i}}}$.

$$CTSFFPWA\left(q_{1},q_{2},\ldots ,q_{n}\right)\leq CTSFFPWA\left({\overset{´}{q}}_{1},{\overset{´}{q}}_{2},\ldots ,\overset{´}{q_{n}}\right)$$

*The proof is****provided****in the appendix*.

Definition 14 *Consider*$q_{i}=\left(v_{i}.\;e^{i2\pi \theta_{v_{i}}},u_{i}.\;e^{i2\pi \varphi_{u_{i}}},w_{i}.\;e^{i2\pi \psi_{w_{i}}}\right)$*be a CTSFFV and WV*$R_{i}={\left(r_{1},r_{2},\ldots ,r_{n}\right)}^{t}$*of*$q_{i}$. *The CTSFFPOWA operator is the function*: $Y^{*n}⟶Y^{*}$, *defined as*:

$$CTSFFPOWA\left(q_{1},q_{2},\ldots ,q_{n}\right)={⊕}_{i=1}^{n}\left(q_{s\left(i\right)}R_{i}\right)$$

*In this*, $\left(q_{1},q_{2},\ldots ,q_{n}\right)$*are the collection of permutations such as*$q_{i-1}\geq q_{i}$. *On CTSFFVs*, *apply frank operational laws and detail the following theorem given below*:

Theorem 6 Consider $q_{i}=\left(v_{i}.\;e^{i2\pi \theta_{v_{i}}},u_{i}.\;e^{i2\pi \varphi_{u_{i}}},w_{i}.\;e^{i2\pi \psi_{w_{i}}}\right)$ be a CTSFFV. So, aggregate the values of $\overset{´}{q_{i\left(s\right)}}$ using the CTSFFPOWA and CTSFFV are presented in Equation (19).

(19) $$CTSFFPOWA\left(q_{1},q_{2},\ldots ,q_{n}\right)=\left(\begin{array}{c} \sqrt[{t}]{1-\log_{o}\left(1+\prod_{i=1}^{n}{\left(\left(o^{1-{\left(v_{s\left(i\right)}\right)}^{t}}-1\right)\right)}^{R_{i}}\right)},e^{i2\pi \sqrt[{t}]{1-\log_{o}\left(1+\prod_{i=1}^{n}{\left(\left(o^{1-{\left(\theta_{v_{s\left(i\right)}}\right)}^{t}}-1\right)\right)}^{R_{i}}\right)},}\\ \log_{o}\left(1+\prod_{i=1}^{n}{\left(\left(o^{{\left(u_{s\left(i\right)}\right)}^{t}}-1\right)\right)}^{R_{i}}\right),e^{i2\pi \;\log_{o}\left(1+\prod_{i=1}^{n}{\left(\left(o^{{\left(\varphi_{u_{s\left(i\right)}}\right)}^{t}}-1\right)\right)}^{R_{i}}\right)}\\ \log_{o}\left(1+\prod_{i=1}^{n}{\left(\left(o^{{\left(w_{s\left(i\right)}\right)}^{t}}-1\right)\right)}^{R_{i}}\right),e^{i2\pi \;\log_{o}\left(1+\prod_{i=1}^{n}{\left(\left(o^{{\left(\psi_{w_{s\left(i\right)}}\right)}^{t}}-1\right)\right)}^{R_{i}}\right)} \end{array}\right)$$

Definition 15 *Consider*$q_{i}$*to be the group of CTSFFVs*. *A CTSFFPHWA operator of dimension*n*is the mapping*$CTSFFPHWA:y^{*n}⟶y^{*}$as

$$CTSFFPHWA\left(q_{1},q_{2},\ldots ,q_{n}\right)={⊕}_{i=1}^{n}\left(\overset{´}{q_{i}}R_{i}\right)$$

*In this WV*, *the CTSFFPHWA operator is*$R_{i}={\left(r_{1},r_{2},\ldots ,r_{n}\right)}^{t}$*having a condition as*$R_{I}=\left[0,1\right]$*and*$\sum_{i=1}^{n}R_{i}=1$; $\overset{´}{\overset{´}{q_{i}}=n\overset{´}{q_{i}}R_{i}}$, $\left({\overset{´}{q}}_{1},{\overset{´}{q}}_{2},\ldots ,\overset{´}{q_{n}}\right)$*is the permutation of the collection of weight CTSFFVs*, *such as*$\overset{´}{q_{i-1}}=\overset{´}{q_{i}}\;\forall i$; $R_{i}={\left(r_{1},r_{2},\ldots ,r_{n}\right)}^{t}$. *The factor*n, *is responsible for carrying equilibrium*.

Theorem 7 *Consider*$q_{i}=\left(v_{i}.\;e^{i2\pi \theta_{v_{i}}},u_{i}.\;e^{i2\pi \varphi_{u_{i}}},w_{i}.\;e^{i2\pi \psi_{w_{i}}}\right)$*be a CTSFFV*. *Then*, *aggregate the values of*$\overset{´}{q_{i}}s$. *Applying CTSFFPHWA operation further gives CTSFFVs*.

$$CTSFFPHWA\left(q_{1},q_{2},\ldots ,q_{n}\right)={⊕}_{i=1}^{n}\left(\overset{´}{q_{i}}R_{i}\right)$$

*Next*,

$$CTSFFPHWA\left(q_{1},q_{2},\ldots ,q_{n}\right)=\left(\begin{array}{c} \sqrt[{t}]{1-\log_{o}\left(1+\prod_{i=1}^{n}{\left(\left(o^{1-{\left(\overset{´}{v_{i}}\right)}^{t}}-1\right)\right)}^{R_{i}}\right)},e^{i2\pi \sqrt[{t}]{1-\log_{o}\left(1+\prod_{i=1}^{n}{\left(\left(o^{1-{\left(\overset{´}{\theta_{v_{i}}}\right)}^{t}}-1\right)\right)}^{R_{i}}\right)},}\\ \log_{o}\left(1+\prod_{i=1}^{n}{\left(\left(o^{{\left(\overset{´}{u_{i}}\right)}^{t}}-1\right)\right)}^{R_{i}}\right),e^{i2\pi \;\log_{o}\left(1+\prod_{i=1}^{n}{\left(\left(o^{{\left(\overset{´}{\varphi_{u_{i}}}\right)}^{t}}-1\right)\right)}^{R_{i}}\right)}\\ \log_{o}\left(1+\prod_{i=1}^{n}{\left(\left(o^{{\left({\overset{´}{w}}_{i}\right)}^{t}}-1\right)\right)}^{R_{i}}\right),e^{i2\pi \;\log_{o}\left(1+\prod_{i=1}^{n}{\left(\left(o^{{\left(\overset{´}{\psi_{w_{i}}}\right)}^{t}}-1\right)\right)}^{R_{i}}\right)} \end{array}\right)$$

The proof of this theorem is the same as Theorem 2.

Definition 16 *Let*$q_{i}=\left(v_{i}.\;e^{i2\pi \theta_{v_{i}}},u_{i}.\;e^{i2\pi \varphi_{u_{i}}},w_{i}.\;e^{i2\pi \psi_{w_{i}}}\right)$*be a CTSFV and*$R_{i}={\left(r_{1},r_{2},\ldots ,r_{n}\right)}^{t}$*is a WV of*$q_{i}$, *having condition*$R_{i}\geq 0$*and*$\sum_{i=1}^{n}R_{i}=1$. *Therefore*, *the CTSFFPWG operator has a function*: $y^{*n}⟶y^{*}$, *can be defined as*:

$$CTSFFPWG\left(q_{1},q_{2},\ldots ,q_{n}\right)={⊗}_{i=1}^{n}\left(R_{i}^{t_{i}}\right)$$

*By using frank operations on CTSFFVs*. *We also obtain the following theorem*.

Theorem 8 *Suppose*$q_{i}=\left(v_{i}.\;e^{i2\pi \theta_{v_{i}}},u_{i}.\;e^{i2\pi \varphi_{u_{i}}},w_{i}.\;e^{i2\pi \psi_{w_{i}}}\right)$*is a CTSFFV*. *Using CTSFFPWG AOs and aggregating the information of*$q_{i}$, *then obtaining results given in* Equation (20) *are also CTSFFVs*.

(20) $$CTSFFPWG\left(q_{1},q_{2},\ldots ,q_{n}\right)={⊗}_{i=1}^{n}\left(R_{i}^{t_{i}}\right)=\left(\begin{array}{c} \log_{o}\left(1+\prod_{i=1}^{n}\left({\left(o^{v_{i}^{t}}-1\right)}^{R_{i}}\right)\right),e^{i2\pi \;\log_{o}\left(1+\prod_{i=1}^{n}\left({\left(o^{{\left(\theta_{v_{i}}\right)}^{t}}-1\right)}^{R_{i}}\right)\right)}\\ \sqrt[{t}]{1-\log_{o}\left(1+\prod_{i=1}^{n}{\left(\left(o^{1-u_{i}^{t}}-1\right)\;\right)}^{R_{i}}\;\right)},e^{i2\pi \sqrt[{t}]{1-\log_{o}\left(1+\prod_{i=1}^{n}{\left(\left(o^{1-{\left(\varphi_{u_{i}}\right)}^{t}}-1\right)\;\right)}^{R_{i}}\right)}}\\ \sqrt[{t}]{1-\log_{o}\left(1+\prod_{i=1}^{n}{\left(\left(o^{1-w_{i}^{t}}-1\right)\;\right)}^{R_{i}}\;\right)},e^{i2\pi \sqrt[{t}]{1-\log_{o}\left(1+\prod_{i=1}^{n}{\left(\left(o^{1-{\left(\psi_{w_{i}}\right)}^{t}}-1\right)\;\right)}^{R_{i}}\right)}} \end{array}\right)$$

*The proof is****provided****in the appendix*.

Definition 17 Consider $q_{i}=\left(v_{i}.\;e^{i2\pi \theta_{v_{i}}},u_{i}.\;e^{i2\pi \varphi_{u_{i}}},w_{i}.\;e^{i2\pi \psi_{w_{i}}}\right)$ be the group of CTSFFVs. $R_{i}={\left(r_{1},r_{2},\ldots ,r_{n}\right)}^{t}$ be the WV, such as $R_{i}\geq 0\;\text{and}\;\sum_{i=1}^{n}R_{i}=1$. A CPTSFFOWG operator of size n is a mapping $\text{CTSFFPOWG}:y^{*n}⟶y^{*}$.

$$CTSFFPOWG\left(q_{1},q_{2},\ldots ,q_{n}\right)={⊗}_{i=1}^{n}\left(R_{i}^{t_{s\left(i\right)}}\right)$$

Theorem 9 *Consider*$q_{i}=\left(v_{i}.\;e^{i2\pi \theta_{v_{i}}},u_{i}.\;e^{i2\pi \varphi_{u_{i}}},w_{i}.\;e^{i2\pi \psi_{w_{i}}}\right)$*is a CTSFFV*. *So*, *aggregating the values of*$\overset{´}{q_{i}}$*s using the CTSFFPOWG also gives CTSFFV*:

(21) $$CTSFFPOWG\left(q_{1},q_{2},\ldots ,q_{n}\right)=\left(\begin{array}{c} \log_{o}\left(1+\prod_{i=1}^{n}\left({\left(o^{v_{s\left(i\right)}^{t}}-1\right)}^{R_{i}}\right)\right),e^{i2\pi \;\log_{o}\left(1+\prod_{i=1}^{n}\left({\left(o^{{\left(\theta_{v_{s\left(i\right)}}\right)}^{t}}-1\right)}^{R_{i}}\right)\right)}\\ \sqrt[{t}]{1-\log_{o}\left(1+\prod_{i=1}^{n}{\left(\left(o^{1-u_{s\left(i\right)}^{t}}-1\right)\;\right)}^{R_{i}}\;\right)},e^{i2\pi \sqrt[{t}]{1-\log_{o}\left(1+\prod_{i=1}^{n}{\left(\left(o^{1-{\left(\varphi_{u_{s\left(i\right)}}\right)}^{t}}-1\right)\;\right)}^{R_{i}}\right)}}\\ \sqrt[{t}]{1-\log_{o}\left(1+\prod_{i=1}^{n}{\left(\left(o^{1-w_{s\left(i\right)}^{t}}-1\right)\;\right)}^{R_{i}}\;\right)},e^{i2\pi \sqrt[{t}]{1-\log_{o}\left(1+\prod_{i=1}^{n}{\left(\left(o^{1-{\left(\psi_{w_{s\left(i\right)}}\right)}^{t}}-1\right)\;\right)}^{R_{i}}\right)}} \end{array}\right)$$

Definition 18 *Consider*$q_{i}$*be to the group of CTSFFVs*. *A CTSFFPHWG operator of dimension*n*is the mapping*$CTSFFPHWG:y^{*n}⟶y^{*}$as

$$CTSFFPHWG\left(q_{1},q_{2},\ldots ,q_{n}\right)={⊗}_{i=1}^{n}\left(R_{i}^{\overset{´}{t_{i}}}\right)$$

*In this WV*, *the CTSFFPHWG operator is*$R_{i}={\left(r_{1},r_{2},\ldots ,r_{n}\right)}^{t}$*having a situation as*$R_{i}=\left[0,1\right]$*and*$\sum_{i=1}^{n}R_{i}=1$; $\overset{´}{\overset{´}{q_{i}}=n\overset{´}{q_{i}}R_{i}}$, $\left({\overset{´}{q}}_{1},{\overset{´}{q}}_{2},\ldots ,\overset{´}{q_{n}}\right)$*is the permutation of the collection of weight CTSFFVs*, *such as*$\overset{´}{q_{i-1}}=\overset{´}{q_{i}}\;\forall i$; $R_{i}={\left(r_{1},r_{2},\ldots ,r_{n}\right)}^{t}$. *The factor*n, *is responsible for carrying equilibrium*.

Theorem 10 Consider $q_{i}=\left(v_{i}.\;e^{i2\pi \theta_{v_{i}}},u_{i}.\;e^{i2\pi \varphi_{u_{i}}},w_{i}.\;e^{i2\pi \psi_{w_{i}}}\right)$ is a CTSFFV. Then, aggregate the values of $\overset{´}{q_{i\left(s\right)}}$. Applying CTSFFPHWG operation further gives CTSFFVs and results are presented in Equation in (21).

(22) $$CTSFFPWG\left(q_{1},q_{2},\ldots ,q_{n}\right)={⊗}_{i=1}^{n}\left(R_{i}^{t_{i}}\right)=\left(\begin{array}{c} \log_{o}\left(1+\prod_{i=1}^{n}\left({\left(o^{\overset{´}{v_{i}^{t}}}-1\right)}^{R_{i}}\right)\right),e^{i2\pi \;\log_{o}\left(1+\prod_{i=1}^{n}\left({\left(o^{{\left(\overset{´}{\theta_{v_{i}}}\right)}^{t}}-1\right)}^{R_{i}}\right)\right)}\\ \sqrt[{t}]{1-\log_{o}\left(1+\prod_{i=1}^{n}{\left(\left(o^{1-\overset{´}{u_{i}^{t}}}-1\right)\;\right)}^{R_{i}}\;\right)},e^{i2\pi \sqrt[{t}]{1-\log_{o}\left(1+\prod_{i=1}^{n}{\left(\left(o^{1-{\left(\overset{´}{\varphi_{u_{i}}}\right)}^{t}}-1\right)\;\right)}^{R_{i}}\right)}}\\ \sqrt[{t}]{1-\log_{o}\left(1+\prod_{i=1}^{n}{\left(\left(o^{1-\overset{´}{w_{i}^{t}}}-1\right)\;\right)}^{R_{i}}\;\right)},e^{i2\pi \sqrt[{t}]{1-\log_{o}\left(1+\prod_{i=1}^{n}{\left(\left(o^{1-{\left(\overset{´}{\psi_{w_{i}}}\right)}^{t}}-1\right)\;\right)}^{R_{i}}\right)}} \end{array}\right)$$

## 6 MADM algorithm based on CTSFFPWA and CTSFFPWG operators

In this segment, we aggregate the data using proposed TSFFPWA and CTSFFPWG operators under the CTSFVs environment. For this, let $Ħ=\left({Ħ}_{1},{Ħ}_{2},\ldots ,{Ħ}_{k}\right)$ are k distinct possible alternatives for selection. Suppose $d=\left\{d_{1},d_{2},\ldots ,d_{t}\right\}$ be attributes with WV R with condition $\sum_{i=1}^{n}R_{i}=1$. Consider complex prioritized TSF (CPTSF) data $M={\left(h_{ij}\right)}_{m\times n}$ in the shape of a matrix where CTSFPVs $q_{ij}=\left(v_{i}.\;e^{i2\pi \theta_{v_{i}}},u_{i}.\;e^{i2\pi \varphi_{u_{i}}},w_{i}.\;e^{i2\pi \psi_{w_{i}}}\right)$ shows the value of the attributes and fulfilled Condition $0\leq \left(v_{\text{ij}}.\;e^{i2\pi \theta_{v_{\text{ij}}}},u_{\text{ij}}.\;e^{i2\pi \varphi_{u_{\text{ij}}}},w_{\text{ij}}.\;e^{i2\pi \psi_{w_{\text{ij}}}}\right)\leq 1$. Finally, construct the CTSF decision matrix $M={\left(h_{ij}\right)}_{m\times n}$ usnig CTSFS information.

In this article, to select the best choice, apply the developer approach CTSFFPWA and CTSFFPWG AOs to solve the problem based on MADM methodology in the CTSFS environment. The algorithm of the proposed technique is given as follows.

Step 1 Data investigation in the form of CTSFVs and normalization of the decision matrix.
Step 2 Construct the CTSF decision matrix $M={\left(h_{ij}\right)}_{m\times n}$.
Step 3 Using the operation of CTSFFPWA and CTSFFPWG operators with prioritized WV to aggregate CTSF information:

$$\text{CTSFFPWA}\left(q_{1},q_{2},\ldots ,q_{n}\right)={⊕}_{i=1}^{n}\left(t_{i}R_{i}\right)=\left(\begin{array}{c} \sqrt[{t}]{1-\log_{o}\left(1+\prod_{i=1}^{n}{\left(\left(o^{1-{\left(v_{i}\right)}^{t}}-1\right)\right)}^{R_{i}}\right)},e^{i2\pi \sqrt[{t}]{1-\log_{o}\left(1+\prod_{i=1}^{n}{\left(\left(o^{1-{\left(\theta_{v_{i}}\right)}^{t}}-1\right)\right)}^{R_{i}}\right)},}\\ \log_{o}\left(1+\prod_{i=1}^{n}{\left(\left(o^{{\left(u_{i}\right)}^{t}}-1\right)\right)}^{R_{i}}\right),e^{i2\pi \left(\log_{o}\left(1+\prod_{i=1}^{n}{\left(\left(o^{{\left(\varphi_{u_{i}}\right)}^{t}}-1\right)\right)}^{R_{i}}\right)\right)}\\ \log_{o}\left(1+\prod_{i=1}^{n}{\left(\left(o^{{\left(w_{i}\right)}^{t}}-1\right)\right)}^{R_{i}}\right),e^{i2\pi \left(\log_{o}\left(1+\prod_{i=1}^{n}{\left(\left(o^{{\left(\psi_{w_{i}}\right)}^{t}}-1\right)\right)}^{R_{i}}\right)\right)} \end{array}\right)$$

$$CTSFFPWG\left(q_{1},q_{2},\ldots ,q_{n}\right)={⊗}_{i=1}^{n}\left(R_{i}^{t_{i}}\right)=\left(\begin{array}{c} \log_{o}\left(1+\prod_{i=1}^{n}\left({\left(o^{v_{i}^{t}}-1\right)}^{R_{i}}\right)\right),e^{i2\pi \left(\log_{o}\left(1+\prod_{i=1}^{n}\left({\left(o^{{\left(\theta_{v_{i}}\right)}^{t}}-1\right)}^{R_{i}}\right)\right)\right)}\\ \sqrt[{t}]{1-\log_{o}\left(1+\prod_{i=1}^{n}{\left(\left(o^{1-u_{i}^{t}}-1\right)\;\right)}^{R_{i}}\;\right)},e^{i2\pi \sqrt[{t}]{1-\log_{o}\left(1+\prod_{i=1}^{n}{\left(\left(o^{1-{\left(\varphi_{u_{i}}\right)}^{t}}-1\right)\;\right)}^{R_{i}}\right)}}\\ \sqrt[{t}]{1-\log_{o}\left(1+\prod_{i=1}^{n}{\left(\left(o^{1-w_{i}^{t}}-1\right)\;\right)}^{R_{i}}\;\right)},e^{i2\pi \sqrt[{t}]{1-\log_{o}\left(1+\prod_{i=1}^{n}{\left(\left(o^{1-{\left(\psi_{w_{i}}\right)}^{t}}-1\right)\;\right)}^{R_{i}}\right)}} \end{array}\right)$$

Step 4 In this step, find the alternatives' SVs and construct a ranking order.

$$S\left({Ѣ}_{i}\right)=\frac{2+\left[\left(v_{Ѣ}\left(y\right)-u_{Ѣ}\left(y\right)-w\left(y\right)\right)+\left(\theta_{v_{Ѣ}\left(y\right)}-\varphi_{u_{Ѣ}\left(y\right)}-\psi_{w_{Ѣ}\left(y\right)}\right)\right]}{4}$$

Step 5 Arrange all alternatives using the ranking values.
Step 6 If the score value for one alternative is maximum, then this would be the most preferable, i.e., the best. Otherwise, the accuracy function value should be calculated for all the options with the same SVs.
• This would be the best alternative if the AV is maximum for only one of those alternatives.
• If the AV is maximum for two or more of those alternatives, then any of them could be selected as the best alternative.

**Fig. 1:**
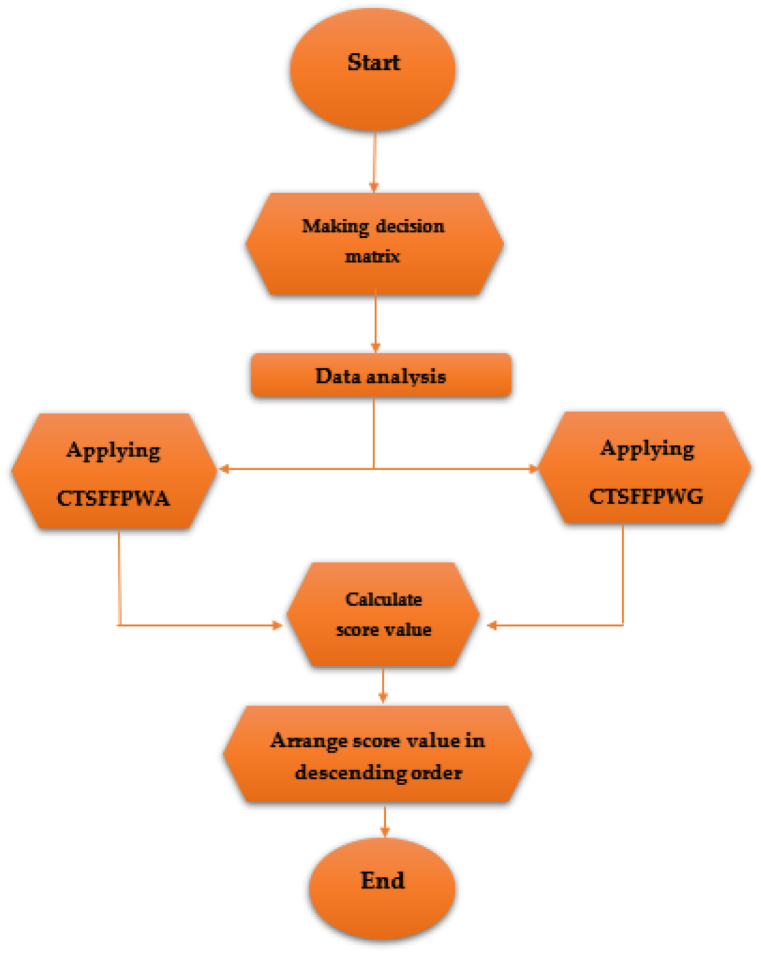
The proposed algorithm is based on the TSFVs.

The proposed MADM algorithm in the shape of folw chart based on the daignosed theory is presented in Fig. 1.

## 7 Numerical example

This article solves the MADM problem using the CTSFFPWA and CTSFFPWG operators. In this example, our objective is to discuss the solar system selection problem using the proposed AOs and choose the best solar system. A Solar system plays a vital role in the production of electricity. Nowadays, the discussion of energy resources is a hot discussion topic worldwide. These days, the idea of the solar system for electricity production is a trending topic. The solar system uses sunlight for electricity production, and the sun's heat has been extensively used to give electricity to factories and houses through solar systems. There are many applications of the solar system in our daily lives, so the significance of the solar system is not ignorable.

**Fig. 2:**
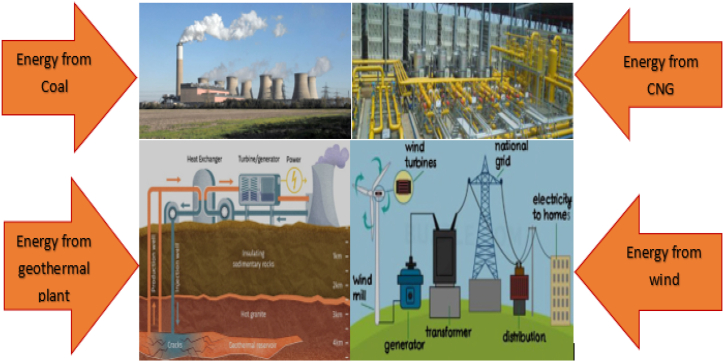
Shows the different sources of electricity.

There are many sources of electricity, such as fossil fuels, oil, condensed natural gas (CNG), nuclear power plants, wind power, and geothermal power plants. These discussed energy sources produce a reliable and reasonable amount of electricity and fulfill the electricity demand, but on the other hand, they have many side effects. For example, due to the combination process, fossil fuels produce a bulk of smoke, which causes harmful climate changes for human beings. While electricity produced through nuclear power plants is costly and dangerous because there is no space for making any small mistake during chemical reactions, it is safe until it is controlled. If it is uncontrolled, it produces unbearable heat, which causes a bomb blast. It is also challenging to destroy nuclear elements after making the required electricity. The CNG is also not a more reasonable and permanent source of electricity because all over the world, the storage of CNG will be finished day by day. The importance of multiple electricity sources can be illustrated through Fig. 2.

The primary source of Energy on Earth is the sun. The solar system is a renewable source. This source can be used repeatedly which can never be exhausted. It has a low cost for this operation. In this world, every person uses an easy way to gain electricity. So, the solar energy system is a good source of energy. It can not affect the habitation around its harnessing area. It is an easy way to gain cheap electricity and a system that does not require any cost for producing electricity from the sun.

**Example 2.** Assume that a person wants to choose the best system from the four different types of solar systems, such as ${Ǽ}_{j}=\left(j=1,2,3,4\right)$. Such as ${Ǽ}_{1}$ is a concentrated Photovoltaic (PV) system, ${Ǽ}_{2}$ is an amorphous system, ${Ǽ}_{3}$ is a polycrystalline system and ${Ǽ}_{4}$ is monocrystalline. The attribute during selection is power output, it's essential to choose a solar panel with a proper power rating to give your energy desires and efficiency, Greater efficiency panels will make more power per unit area and are primarily extra desirable. Temperature: Panels with lower temperature coefficients perform better in hot climates, size, and space availability selected panels that are suitable and fit on your roof and give the necessary power output. Also, keep in mind the following WVs of attributes during the selection process such as ${Ǽ}_{j}=\left(j=1,2,3,4\right)$. We assign weights such as $\alpha_{1}$ is the power output (0.8235), $\alpha_{2}$ is energy efficiency (0.1544), $\alpha_{3}$ is temerature (0.0208), $\alpha_{4}$ is size and space availability (0.0013). All these WVs are calculated by using the concept of priority degree. For a more precise understanding of the decision matrix alternatives ${Ǽ}_{j}=\left(j=1,2,3,4\right)$ are represented column-wise, and information on attributes ${Ǽ}_{j}=\left(j=1,2,3,4\right)$ is given row-wise in Table 2. Then, aggregate data using proposed CTSFFPWA and CTSFFPWG AOs. Where we use parameters t=4 and o=2.

**Table 2:** CTSF decision matrix.

	${Ǽ}_{1}$	${Ǽ}_{2}$	${Ǽ}_{3}$	${Ǽ}_{4}$
$\alpha_{1}$	$\left(\begin{array}{c} \left(0.69,0.5\right)\text{,}\\ \left(0.65,0.85\right),\left(0.36,0.58\right) \end{array}\right)$	$\left(\begin{array}{c} \left(0.52,0.56\right)\text{,}\\ \left(0.56,0.86\right),\left(0.93,0.52\right) \end{array}\right)$	$\left(\begin{array}{c} \left(0.25,0.56\right),\\ \left(0.58,0.38\right),\left(0.86,0.74\right) \end{array}\right)$	$\left(\begin{array}{c} \left(0.58,0.69\right),\\ \left(0.56,0.13\right),\left(0.16,0.82\right) \end{array}\right)$
$\alpha_{2}$	$\left(\begin{array}{c} \left(0.25,0.56\right)\text{,}\\ \left(0.2,0.52\right),\left(0.98,0.28\right) \end{array}\right)$	$\left(\begin{array}{c} \left(0.95,0.85\right)\text{,}\\ \left(0.68,0.25\right),\left(0.96,0.3\right) \end{array}\right)$	$\left(\begin{array}{c} \left(0.8,0.7\right),\\ \left(0.65,0.74\right),\left(0.52,0.65\right) \end{array}\right)$	$\left(\begin{array}{c} \left(0.19,0.64\right),\\ \left(0.58,0.25\right),\left(0.17,0.36\right) \end{array}\right)$
$\alpha_{3}$	$\left(\begin{array}{c} \left(0.52,0.38\right),\\ \left(0.25,0.85\right),\left(0.65,0.4\right) \end{array}\right)$	$\left(\begin{array}{c} \left(0.23,0.58\right)\text{,}\\ \left(0.29,0.52\right),\left(0.45,0.63\right) \end{array}\right)$	$\left(\begin{array}{c} \left(0.85,0.51\right)\text{,}\\ \left(0.64,0.34\right),\left(0.54,0.31\right) \end{array}\right)$	$\left(\begin{array}{c} \left(0.45,0.58\right),\\ \left(0.53,0.41\right),\left(0.98,0.28\right) \end{array}\right)$
$\alpha_{4}$	$\left(\begin{array}{c} \left(0.35,0.35\right),\\ \left(0.69,0.35\right),\left(0.72,0.81\right) \end{array}\right)$	$\left(\begin{array}{c} \left(0.91,0.36\right),\\ \left(0.29,0.74\right),\left(0.25,0.3\right) \end{array}\right)$	$\left(\begin{array}{c} \left(0.8,0.9\right),\\ \left(0.87,0.65\right),\left(0.81,0.5\right) \end{array}\right)$	$\left(\begin{array}{c} \left(0.34,0.5\right),\\ \left(0.7,0.29\right),\left(0.38,0.45\right) \end{array}\right)$

**Fig. 3:**
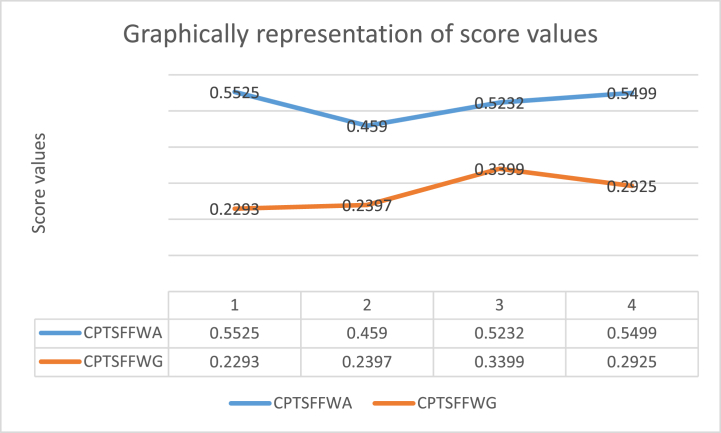
Shows the aggregation ranking of the alternatives using proposed CTSFFPWA and CTSFFPWG operations, where oring lines offer the ranking order using CTSFFPWA AOs. In contrast, the blue line shows the ranking order of alternatives using CTSFFPWG AOs.

For MADM, we first collect information and opinions from experts in Table 2. In this numerical example, we collect information in the form of CTSFVs, including agreements in terms of MG, disagrees in NMG, and assigns as AG in our data in case of absence. Then, we apply our proposed CTSFFPWA and CTSFFPWG operators. The aggregated information is presented in Table 3. And use the score function formula on aggregated, as shown in Table 4., to find the most appropriate option. It also offered a graphical representation of score value for better clarity. At the end, we arrange the alternatives for ranking.

Step 1 First, arrange all the given data in terms of the matrix form provided in Table 2.

Step 2 Aggregate the collection of the decision matrix by using CTSFFPWA and CTSFFPWG operators in Table 3.

Step 3 In this step, calculate the SV using Definition 6. SV of aggregated data is provided in Table 4. The geometrical representation of Table 4 is also given below for a more precise understanding in Fig. 3.

Step 4 In this step, we arrange the data on the base of the score function in Table 5. So ${Ǽ}_{1}$ is the best choice by using CTSFFPWA and using CTSFFPGA ${Ǽ}_{3}$ is the best option.

**Table 3:** Aggregation findings using CTSFFPWA and CTSFFPWG operators.

	CTSFFPWA	CTSFFPWG
${Ǽ}_{1}$	$\left(\begin{array}{c} \left(0.6686,.5127\right)\text{,}\\ \left(0.1613,0.4912\right)\text{,}\\ \left(0.0336,0.1083\right) \end{array}\right)$	$\left(\begin{array}{c} \left(0.1428,0.0727\right)\text{,}\\ \left(0.6274,0.8439\right)\text{,}\\ \left(0.7425,0.5852\right) \end{array}\right)$
${Ǽ}_{2}$	$\left(\begin{array}{c} \left(0.6815,0.6520\right)\text{,}\\ \left(0.0038,0.0480\right)\text{,}\\ \left(0.8652,0.0069\right) \end{array}\right)$	$\left(\begin{array}{c} \left(0.0196,0.1599\right)\text{,}\\ \left(0.5106,0.5076\right)\text{,}\\ \left(0.9720,0.3495\right) \end{array}\right)$
${Ǽ}_{3}$	$\left(\begin{array}{c} \left(0.5221,0.4375\right)\text{,}\\ \left(0.0047,0.3658\right)\text{,}\\ \left(0.1411,0.0333\right) \end{array}\right)$	$\left(\begin{array}{c} \left(0.0353,0.0345\right)\text{,}\\ \left(0.3401,0.7980\right)\text{,}\\ \left(0.6346,0.4933\right) \end{array}\right)$
${Ǽ}_{4}$	$\left(\begin{array}{c} \left(0.6410,0.4306\right)\text{,}\\ \left(0.1383,0.0254\right)\text{,}\\ \left(0.1451,0.2335\right) \end{array}\right)$	$\left(\begin{array}{c} \left(0.0481,0.0188\right)\text{,}\\ \left(0.6647,0.5634\right)\text{,}\\ \left(0.6794,0.7527\right) \end{array}\right)$

**Table 4:** Score values of alternatives using CTSFFPWA and CTSFFPWG operators.

	CTSFFPWA	CTSFFPWG
${Ǽ}_{1}$	0.5525	0.2293
${Ǽ}_{2}$	0.4590	0.2397
${Ǽ}_{3}$	0.5232	0.3399
${Ǽ}_{4}$	0.5499	0.2925

## 8 Comparative study

In this section of the article, study the compiled finding of the scheme CTSFFPWA and CTSFFPWG operator to compare with complex TSF Dombi weighted averaging (CTSFDWA) and weighted geometric (CTSFDWG) by Karaaslan and Al-Husseinawi. [53], CTSF Hamacher (CTSFHWA) and weighted geometric (WG) (CTSFHWG) operator proposed by Ullah et al. [54] and CTSF power weighted Averaging (WA) (CTSFPWA) and CTSF power WG (CTSFPWG) developed by Khan et al. [55] Many other existing AOs such as q-rung orthopair (q-ROF) Aczel Alsina WA (q-ROFAAWA) and q-ROF Aczel-Alsina WG (q-ROFAAWG) operators proposed by Ref. [56] and bipolar soft FS (BSFS) presented by Mahmood [57]. The concept of TSF Dombi WA (TSFDWA) and TSF Dombi WG (TSFDWG) operators provided by Mahmooed et al. [58] and linguistic PyFS (LPyFS) given by Garg [59]. These following AOs cannot deal with such information in which data is two-dimensional and periodic. At the same time, our developed structures, CTSFFPWA and CTSFFPWG operators, can deal with dimensional and periodic information. The comparative analysis of discussed AOs is provided in Table 6. The graphical representation of this analysis is also dipicted in Fig. 3.

**Table 5:** Ordering of alternatives based on SVs.

	Ordering
CTSFFPWA	${Ǽ}_{1}\left(0.5525\right)>{Ǽ}_{4}\left(0.5499\right)>{Ǽ}_{3}\left(0.5232\right)>{Ǽ}_{2}\left(0.4590\right)$
CTSFFPWG	${Ǽ}_{3}\left(0.3399\right)>{Ǽ}_{4}\left(0.3399\right)>{Ǽ}_{2}\left(0.2397\right)>{Ǽ}_{1}\left(0.2293\right)$

Hence, the suitable choice to use CTSFFPWA is ${Ǽ}_{1}$, and to use CTSFFPWG is ${Ǽ}_{3}$.

**Table 6:** Comparative analysis with existing AOs.

Method	Operator	Ranking
Proposed work	CTSFFPWA	${Ǽ}_{1}>{Ǽ}_{4}>{Ǽ}_{3}>{Ǽ}_{2}$
	CTSFFPWG	${Ǽ}_{3}>{Ǽ}_{4}>{Ǽ}_{2}>{Ǽ}_{1}$
Karaaslan and Al-Husseinawi [53].	CTSFDWA	${Ǽ}_{4}>{Ǽ}_{2}>{Ǽ}_{3}>{Ǽ}_{1}$
	CTSFDWG	${Ǽ}_{2}>{Ǽ}_{3}>{Ǽ}_{1}>{Ǽ}_{4}$
Ullah et al. [54]	CTSFHWA	${Ǽ}_{4}>{Ǽ}_{3}>{Ǽ}_{1}>{Ǽ}_{2}$
	CTSFHWG	${Ǽ}_{2}>{Ǽ}_{3}>{Ǽ}_{1}>{Ǽ}_{4}$
Khan et al. [60]	CTSFPWA	${Ǽ}_{4}>{Ǽ}_{3}>{Ǽ}_{1}>{Ǽ}_{2}$
	CTSFPWG	${Ǽ}_{3}>{Ǽ}_{4}>{Ǽ}_{2}>{Ǽ}_{1}$
Khan et al. [56]	q-ROFAAWA q-ROFAAWG	result not found
Mahmood et al. [57]	BSFS	result not found
Garg [59]	LPyFS	result not found

**Fig. 4:**
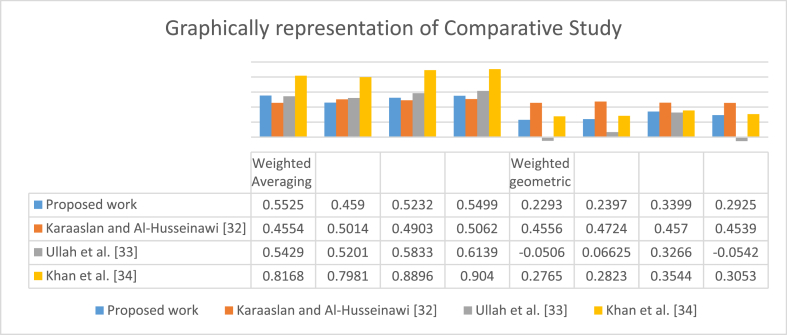
Shows the comparative analysis of aggregation outcomes.

Table 6 provides information about comparing diagnosed AOs with existing techniques and obtaining interesting outcomes, for example, when applying CTSFDWA and CTSFDWG offered by Karaaslan and Al-Husseinawi [53]. on given data, then we get ${Ǽ}_{4}$ and ${Ǽ}_{2}$ be the best alternatives respectively. When applying the CTSFHWA and CTSFHWG proposed by Ullah et al. [54], ${Ǽ}_{4}$ and ${Ǽ}_{2}$ be the best choices respectively. Also, obtained ${Ǽ}_{4}$ and ${Ǽ}_{3}$ be the best options using the CTSFPWA and CTSFPWG operators developed by Khan et al. [60]. On the other hand, many AOs are unable to handle information due to deficiency in their structures, such as q-ROFAAWA and q-ROFAAWG operators presented by Khan et al. [56], BSFS given by Mahmood et al. [57] and LPyFS offered by Garg [59]. We noticed these AOs have no concept of AD with a more excellent range. So that is why they give no results for CTSF information. The graphical representation of the comparative study is discussed in Fig. 4.

It is easily observed from the above pictorial diagram, the comparative analysis of our proposed CTSFFPWA and CTSFFPWG operators with CTSFDWA and CTSFDWG presented by Karaaslan and Al-Husseinawi. [53], CTSFHWA and CTSFHWG were provided by Ullah et al. [54]. The thought of CTSFPWA and CTSFPWG operators is given by Khan et al. [60]. For more clarity, the numeric values of outcomes by applying different AOs can be seen in Fig. 3.

## 9 Results and discussions

In decision-making sciences, the MADM plays a vital role in data aggregation. In crips set theory, the scope of decision-making is limited, but after the innovation of FS theory, the MADM got newborn and achieved the highest peak of popularity.

This portion aims to highlight the significance and importance of proposed CTSFFPWA and CTSFFPWG operators and address the novelty and worth of the TSFS framework. They discussed how much accuracy there is in diagnosed theory than existing one.

The proposed AOs were diagnosed on behalf of FTN and FTCN operational laws. The thought of FTN and FTCN is the most generalized form of existing TNs TCNs. When the decision-maker takes the parameter value as one, the Frank product is changed into the probability product while the Frank sum is transformed into the probability sum. On the other hand, When the decision-maker takes the value of the parameter as infinity, then the Frank product is changed into the Lukasiewicz product. At the same time, the Frank sum is transformed into the Lukasiewicz sum. These significant results enhance the worth of FTN and FTCN operation and give more importance than other existing TNs and TCNs.

The diagnosed CTSFFPWA and CTSFFPWG operators are based on the CTSFS theory. The idea of CTSFS is the most generalized shape of CFS. The remarkable chanKhanges can be seen as follows: The CTSFS is turned into the CFS if AD and NMD are zero, the CTSFS is turned into the CIFS if AD is zero, and the CTSFS is turned into the CSFS if the power of ND, AD, and NMD is one.

The manuscript contains the CTSFFPWA and CTSFFPWG theory and proofs of AOs axioms such as boundedness, idempotency, and monotonicity. The main diagnosed aggregation findings are discussed in Table 4. The graphical representation of the investigated results is presented in Fig. 2. To show the suggested work's worth and accuracy, compare it with existing AOs. The outcomes and graphical representation are discussed in Table 6 and Fig. 3 respectively. During the data aggregation procedure, due to structure limitations, many AOs fail to handle CTSFS information, such as q-ROFAAWA and q-ROFAAWG operators, BSFS, and LPyFS.

The novel findings of the article present the soler system selection problem in numerical examples by applying the proposed CTSFFPWA and CTSFFPWG theory. Because the energy crisis issue is becoming a big joint problem soon, finding different suitable energy resources is a trending problem. The defined methodology is a universal technique for finding the best alternative from multiple options.

### 9.1 Consequences of proposed theory

In the proposed AOs, noticeable features can be observed in CTSFFPWA and CTSFFPWG theory. The presented approach is the generalization of CPFS, addressed by Shanthi et al. [12], and the idea of CSFS, diagnosed by Ali et al. [13].

$$\text{CTSFFPWA}\left(q_{1},q_{2},\ldots ,q_{n}\right)={⊕}_{i=1}^{n}\left(t_{i}R_{i}\right)=\left(\begin{array}{c} \sqrt[{t}]{1-\log_{o}\left(1+\prod_{i=1}^{n}{\left(\left(o^{1-{\left(v_{i}\right)}^{t}}-1\right)\right)}^{R_{i}}\right)},e^{i2\pi \sqrt[{t}]{1-\log_{o}\left(1+\prod_{i=1}^{n}{\left(\left(o^{1-{\left(\theta_{v_{i}}\right)}^{t}}-1\right)\right)}^{R_{i}}\right)},}\\ \log_{o}\left(1+\prod_{i=1}^{n}{\left(\left(o^{{\left(u_{i}\right)}^{t}}-1\right)\right)}^{R_{i}}\right),e^{i2\pi \left(\log_{o}\left(1+\prod_{i=1}^{n}{\left(\left(o^{{\left(\varphi_{u_{i}}\right)}^{t}}-1\right)\right)}^{R_{i}}\right)\right)}\\ \log_{o}\left(1+\prod_{i=1}^{n}{\left(\left(o^{{\left(w_{i}\right)}^{t}}-1\right)\right)}^{R_{i}}\right),e^{i2\pi \left(\log_{o}\left(1+\prod_{i=1}^{n}{\left(\left(o^{{\left(\psi_{w_{i}}\right)}^{t}}-1\right)\right)}^{R_{i}}\right)\right)} \end{array}\right)$$

$$CTSFFPWG\left(q_{1},q_{2},\ldots ,q_{n}\right)={⊗}_{i=1}^{n}\left(R_{i}^{t_{i}}\right)=\left(\begin{array}{c} \log_{o}\left(1+\prod_{i=1}^{n}\left({\left(o^{v_{i}^{t}}-1\right)}^{R_{i}}\right)\right),e^{i2\pi \left(\log_{o}\left(1+\prod_{i=1}^{n}\left({\left(o^{{\left(\theta_{v_{i}}\right)}^{t}}-1\right)}^{R_{i}}\right)\right)\right)}\\ \sqrt[{t}]{1-\log_{o}\left(1+\prod_{i=1}^{n}{\left(\left(o^{1-u_{i}^{t}}-1\right)\;\right)}^{R_{i}}\;\right)},e^{i2\pi \sqrt[{t}]{1-\log_{o}\left(1+\prod_{i=1}^{n}{\left(\left(o^{1-{\left(\varphi_{u_{i}}\right)}^{t}}-1\right)\;\right)}^{R_{i}}\right)}}\\ \sqrt[{t}]{1-\log_{o}\left(1+\prod_{i=1}^{n}{\left(\left(o^{1-w_{i}^{t}}-1\right)\;\right)}^{R_{i}}\;\right)},e^{i2\pi \sqrt[{t}]{1-\log_{o}\left(1+\prod_{i=1}^{n}{\left(\left(o^{1-{\left(\psi_{w_{i}}\right)}^{t}}-1\right)\;\right)}^{R_{i}}\right)}} \end{array}\right)$$

The diagnosed theory is turned into a different fuzzy system by changing the value of parameter t in CTSFFPWA and CTSFFPWG operators.

• When taking t=1, the CTSFFPWA and CTSFFPWG operators reduced into the CPFS.
• When taking t=2, the CTSFFPWA and CTSFFPWG operators reduced into the CSFS.

This remarkable feature shows the superiority and importance of suggested work and provides more accuracy in results than existing approaches.

## 10 Conclusions

MADM is a hot discussion topic in decision-making sciences. So, it is difficult to aggregate information, including two-dimensional and periodic data. Frank's operational laws are among the best choices in this challenging situation. By using the CTSFS theory and idea of prioritized AOs, construct the CTSFFPWA, CTSFFPOWA, CTSFFPHWA, CTSFFPWG, CTSFFPOWG, and CTSFFPHWG operators for CTSFVs data. The CTSFS theory generalizes CIFS, CPyFS, Cq-ROFS, and CPFS theory. In this scenario, some fundamental properties of AOs, such as monotonicity, boundedness, and idempotency. We gave a comprehensive and detailed algorithm based on the proposed CTSFFPWA and CTSFFPWG operators. We also offered a numerical example of solar system selection using the MADM technique and a detailed case study on the importance of solar systems for energy production. Graphical representations of raking results are also part of the manuscript for better clarity. To present the novelty and worth of the diagnosed theory, we construct the comparative analysis with present AOs and provide geometrical representation.

Shortly, our aim is to extend diagnosed work for SFS using Bonferroni AOs presented by Ali et al. [13] and PF Maclaurin symmetric mean (MSM) AOs (PFMSMAOs) proposed by Ullah [61]. Some Dombi operations for complex values are defined by Ali and Mahmood [62], and power AOs for Aczel-Alsina operations based on q-ROFS defined by Khan et al. [63]. Rough IFS prioritized framework defined by Khan et al. [64] Dombi Aos is based on the interval-valued fuzzy information discussed by Seikh and Mandal [65], and interval-valued Dombi Aos is based on unknown weight information provided by Ref. [66]. The frameworks defined in Refs. [[67], [68], [69]] can also be generalized using the developed idea.

## CRediT authorship contribution statement

**Muhammad Rizwan khan:** Writing – original draft, Methodology, Formal analysis, Conceptualization. **Kifayat Ullah:** Writing – original draft, Methodology, Investigation, Formal analysis, Conceptualization. **Ali Raza:** Writing – original draft, Resources, Investigation, Formal analysis, Data curation. **Tapan Senapati:** Writing – review & editing, Supervision, Resources, Investigation. **Sarbast Moslem:** Writing – review & editing, Visualization, Supervision, Methodology.

## Declaration of competing interest

The authors declare that they have no known competing financial interests or personal relationships that could have appeared to influence the work reported in this paper.

## Appendix

**Proof of**Theorem 2**:** We can use induction of the mathematics rule:

$$q_{1}R_{1}=\left(\begin{array}{c} \sqrt[{t}]{1-\log_{o}\left(1+{\left(o^{1-v_{1}^{t}}-1\right)}^{R_{1}}\right)},e^{i2\pi \sqrt[{t}]{1-\log_{o}\left(1+{\left(o^{1-{\left(\theta_{v_{1}}\right)}^{t}}-1\right)}^{R_{1}}\right)}}\\ \log_{o}\left(1+{\left(o^{u_{1}^{t}}-1\right)}^{R_{1}}\right),e^{i2\pi \;\log_{o}\left(1+{\left(o^{{\left(\varphi_{u_{1}}\right)}^{t}}-1\right)}^{R_{1}}\right)}\\ \log_{o}\left(1+{\left(o^{w_{1}^{t}}-1\right)}^{R_{1}}\right),e^{i2\pi \;\log_{o}\left(1+{\left(o^{{\left(\psi_{w_{1}}\right)}^{t}}-1\right)}^{R_{1}}\right)} \end{array}\right)$$

$$q_{2}R_{2}=\left(\begin{array}{c} \sqrt[{t}]{1-\log_{o}\left(1+{\left(o^{1-v_{2}^{t}}-1\right)}^{R_{2}}\right)},e^{i2\pi \sqrt[{t}]{1-\log_{o}\left(1+{\left(o^{1-{\left(\theta_{v_{2}}\right)}^{t}}-1\right)}^{R_{2}}\right)}}\\ \log_{o}\left(1+{\left(o^{u_{2}^{t}}-1\right)}^{R_{2}}\right),e^{i2\pi \;\log_{o}\left(1+{\left(o^{{\left(\varphi_{u_{2}}\right)}^{t}}-1\right)}^{R_{2}}\right)}\\ \log_{o}\left(1+{\left(o^{w_{2}^{t}}-1\right)}^{R_{2}}\right),e^{i2\pi \;\log_{o}\left(1+{\left(o^{{\left(\psi_{w_{2}}\right)}^{t}}-1\right)}^{R_{2}}\right)} \end{array}\right)$$

By using the definition:

$$CTSFFPWA\left(q_{1},q_{2}\right)=q_{1}R_{1}⊕q_{2}R_{2}=\left(\begin{array}{c} \sqrt[{t}]{1-\log_{o}\left(1+{\left(o^{1-v_{1}^{t}}-1\right)}^{R_{1}}\right)},e^{i2\pi \sqrt[{t}]{1-\log_{o}\left(1+{\left(o^{1-{\left(\theta_{v_{1}}\right)}^{t}}-1\right)}^{R_{1}}\right)}}\\ \log_{o}\left(1+{\left(o^{u_{1}^{t}}-1\right)}^{R_{1}}\right),e^{i2\pi \;\log_{o}\left(1+{\left(o^{{\left(\varphi_{u_{1}}\right)}^{t}}-1\right)}^{R_{1}}\right)}\\ \log_{o}\left(1+{\left(o^{w_{1}^{t}}-1\right)}^{R_{1}}\right),e^{i2\pi \;\log_{o}\left(1+{\left(o^{{\left(\psi_{w_{1}}\right)}^{t}}-1\right)}^{R_{1}}\right)} \end{array}\right)⊕\left(\begin{array}{c} \sqrt[{t}]{1-\log_{o}\left(1+{\left(o^{1-v_{2}^{t}}-1\right)}^{R_{2}}\right)},e^{i2\pi \sqrt[{t}]{1-\log_{o}\left(1+{\left(o^{1-{\left(\theta_{v_{2}}\right)}^{t}}-1\right)}^{R_{2}}\right)}}\\ \log_{o}\left(1+{\left(o^{u_{2}^{t}}-1\right)}^{R_{2}}\right),e^{i2\pi \;\log_{o}\left(1+{\left(o^{{\left(\varphi_{u_{2}}\right)}^{t}}-1\right)}^{R_{2}}\right)}\\ \log_{o}\left(1+{\left(o^{w_{2}^{t}}-1\right)}^{R_{2}}\right),e^{i2\pi \;\log_{o}\left(1+{\left(o^{{\left(\psi_{w_{2}}\right)}^{t}}-1\right)}^{R_{2}}\right)} \end{array}\right)$$

$$=\left(\begin{array}{c} \sqrt[{t}]{1-\log_{o}\left(1+{\left(o^{1-v_{1}^{t}}-1\right)}^{R_{1}}{\left(o^{1-v_{2}^{t}}-1\right)}^{R_{2}}\right)},e^{i2\pi \sqrt[{t}]{1-\log_{o}\left(1+{\left(o^{1-{\left(\theta_{v_{1}}\right)}^{t}}-1\right)}^{R_{1}}{\left(o^{1-{\left(\theta_{v_{2}}\right)}^{t}}-1\right)}^{R_{2}}\right)}}\\ \log_{o}\left(1+{\left(o^{u_{1}^{t}}-1\right)}^{R_{1}}{\left(o^{u_{2}^{t}}-1\right)}^{R_{2}}\right),e^{i2\pi \;\log_{o}\left(1+{\left(o^{{\left(\varphi_{u_{1}}\right)}^{t}}-1\right)}^{R_{1}}{\left(o^{{\left(\varphi_{u_{2}}\right)}^{t}}-1\right)}^{R_{2}}\right)}\\ \log_{o}\left(1+{\left(o^{w_{1}^{t}}-1\right)}^{R_{1}}{\left(o^{w_{2}^{t}}-1\right)}^{R_{2}}\right),e^{i2\pi \;\log_{o}\left(1+{\left(o^{{\left(\psi_{w_{1}}\right)}^{t}}-1\right)}^{R_{1}}{\left(o^{{\left(\psi_{w_{2}}\right)}^{t}}-1\right)}^{R_{2}}\right)} \end{array}\right)$$

$$=\left(\begin{array}{c} \sqrt[{t}]{1-\log_{o}\left(1+\prod_{i=1}^{2}{\left(\left(o^{1-v_{i}^{t}}-1\right)\;\right)}^{R_{i}}\;\right)},e^{i2\pi \sqrt[{t}]{1-\log_{o}\left(1+\prod_{i=1}^{2}{\left(\left(o^{1-{\left(\theta_{v_{i}}\right)}^{t}}-1\right)\;\right)}^{R_{i}}\right)}}\\ \log_{o}\left(1+\prod_{i=1}^{2}\left({\left(o^{u_{i}^{t}}-1\right)}^{R_{i}}\right)\right),e^{i2\pi \;\log_{o}\left(1+\prod_{i=1}^{2}\left({\left(o^{{\left(\varphi_{u_{i}}\right)}^{t}}-1\right)}^{R_{i}}\right)\right)}\\ \log_{o}\left(1+\prod_{i=1}^{2}\left({\left(o^{w_{i}^{t}}-1\right)}^{R_{i}}\right)\right),e^{i2\pi \;\log_{o}\left(1+\prod_{i=1}^{2}\left({\left(o^{{\left(\psi_{w_{i}}\right)}^{t}}-1\right)}^{R_{i}}\right)\right)} \end{array}\right)$$

For n=k.

$$=\left(\begin{array}{c} \sqrt[{t}]{1-\log_{o}\left(1+\prod_{i=1}^{k}{\left(\left(o^{1-v_{i}^{t}}-1\right)\;\right)}^{R_{i}}\;\right)},e^{i2\pi \sqrt[{t}]{1-\log_{o}\left(1+\prod_{i=1}^{k}{\left(\left(o^{1-{\left(\theta_{v_{i}}\right)}^{t}}-1\right)\;\right)}^{R_{i}}\right)}}\\ \log_{o}\left(1+\prod_{i=1}^{k}\left({\left(o^{u_{i}^{t}}-1\right)}^{R_{i}}\right)\right),e^{i2\pi \;\log_{o}\left(1+\prod_{i=1}^{k}\left({\left(o^{{\left(\varphi_{u_{i}}\right)}^{t}}-1\right)}^{R_{i}}\right)\right)}\\ \log_{o}\left(1+\prod_{i=1}^{k}\left({\left(o^{w_{i}^{t}}-1\right)}^{R_{i}}\right)\right),e^{i2\pi \;\log_{o}\left(1+\prod_{i=1}^{k}\left({\left(o^{{\left(\psi_{w_{i}}\right)}^{t}}-1\right)}^{R_{i}}\right)\right)} \end{array}\right)$$

For n=k+1.

$$=\left(\begin{array}{c} \sqrt[{t}]{1-\log_{o}\left(1+\prod_{i=1}^{k}{\left(\left(o^{1-v_{i}^{t}}-1\right)\;\right)}^{R_{i}}\;\right)},e^{i2\pi \sqrt[{t}]{1-\log_{o}\left(1+\prod_{i=1}^{k}{\left(\left(o^{1-{\left(\theta_{v_{i}}\right)}^{t}}-1\right)\;\right)}^{R_{i}}\right)}}\\ \log_{o}\left(1+\prod_{i=1}^{k}\left({\left(o^{u_{i}^{t}}-1\right)}^{R_{i}}\right)\right),e^{i2\pi \;\log_{o}\left(1+\prod_{i=1}^{k}\left({\left(o^{{\left(\varphi_{u_{i}}\right)}^{t}}-1\right)}^{R_{i}}\right)\right)}\\ \log_{o}\left(1+\prod_{i=1}^{k}\left({\left(o^{w_{i}^{t}}-1\right)}^{R_{i}}\right)\right),e^{i2\pi \;\log_{o}\left(1+\prod_{i=1}^{k}\left({\left(o^{{\left(\psi_{w_{i}}\right)}^{t}}-1\right)}^{R_{i}}\right)\right)} \end{array}\right)⊕\left(\begin{array}{c} \sqrt[{t}]{1-\log_{o}\left(1+\prod_{i=1}^{k+1}{\left(\left(o^{1-v_{i}^{t}}-1\right)\;\right)}^{R_{i}}\;\right)},e^{i2\pi \sqrt[{t}]{1-\log_{o}\left(1+\prod_{i=1}^{k+1}{\left(\left(o^{1-{\left(\theta_{v_{i}}\right)}^{t}}-1\right)\;\right)}^{R_{i}}\right)}}\\ \log_{o}\left(1+\prod_{i=1}^{k+1}\left({\left(o^{u_{i}^{t}}-1\right)}^{R_{i}}\right)\right),e^{i2\pi \;\log_{o}\left(1+\prod_{i=1}^{k+1}\left({\left(o^{{\left(\varphi_{u_{i}}\right)}^{t}}-1\right)}^{R_{i}}\right)\right)}\\ \log_{o}\left(1+\prod_{i=1}^{k+1}\left({\left(o^{w_{i}^{t}}-1\right)}^{R_{i}}\right)\right),e^{i2\pi \;\log_{o}\left(1+\prod_{i=1}^{k+1}\left({\left(o^{{\left(\psi_{w_{i}}\right)}^{t}}-1\right)}^{R_{i}}\right)\right)} \end{array}\right)$$

$$=\left(\begin{array}{c} \sqrt[{t}]{1-\log_{o}\left(1+\prod_{i=1}^{k+1}{\left(\left(o^{1-v_{i}^{t}}-1\right)\;\right)}^{R_{i}}\;\right)},e^{i2\pi \sqrt[{t}]{1-\log_{o}\left(1+\prod_{i=1}^{k+1}{\left(\left(o^{1-{\left(\theta_{v_{i}}\right)}^{t}}-1\right)\;\right)}^{R_{i}}\right)}}\\ \log_{o}\left(1+\prod_{i=1}^{k+1}\left({\left(o^{u_{i}^{t}}-1\right)}^{R_{i}}\right)\right),e^{i2\pi \;\log_{o}\left(1+\prod_{i=1}^{k+1}\left({\left(o^{{\left(\varphi_{u_{i}}\right)}^{t}}-1\right)}^{R_{i}}\right)\right)}\\ \log_{o}\left(1+\prod_{i=1}^{k+1}\left({\left(o^{w_{i}^{t}}-1\right)}^{R_{i}}\right)\right),e^{i2\pi \;\log_{o}\left(1+\prod_{i=1}^{k+1}\left({\left(o^{{\left(\psi_{w_{i}}\right)}^{t}}-1\right)}^{R_{i}}\right)\right)} \end{array}\right)$$

So, this expression is true for n=k+1.

**Proof of**Theorem 3**:** Consider $q_{i}=\left(v_{i}.\;e^{i2\pi \theta_{v_{i}}},u_{i}.\;e^{i2\pi \varphi_{u_{i}}},w_{i}.\;e^{i2\pi \psi_{w_{i}}}\right)$; (i=1,2,…,z).

$$CTSFFPWA\left(q_{1},q_{2},\ldots ,q_{n}\right)={⊕}_{i=1}^{n}=\left(\begin{array}{c} \sqrt[{t}]{1-\log_{o}\left(1+\prod_{i=1}^{z}{\left(\left(o^{1-{\left(v_{i}\right)}^{t}}-1\right)\right)}^{R_{i}}\right)},e^{i2\pi \sqrt[{t}]{1-\log_{o}\left(1+\prod_{i=1}^{z}{\left(\left(o^{1-{\left(\theta_{v_{i}}\right)}^{t}}-1\right)\right)}^{R_{i}}\right)},}\\ \log_{o}\left(1+\prod_{i=1}^{z}{\left(\left(o^{{\left(u_{i}\right)}^{t}}-1\right)\right)}^{R_{i}}\right),e^{i2\pi \;\log_{o}\left(1+\prod_{i=1}^{z}{\left(\left(o^{{\left(\varphi_{u_{i}}\right)}^{t}}-1\right)\right)}^{R_{i}}\right)}\\ \log_{o}\left(1+\prod_{i=1}^{z}{\left(\left(o^{{\left(w_{i}\right)}^{t}}-1\right)\right)}^{R_{i}}\right),e^{i2\pi \;\log_{o}\left(1+\prod_{i=1}^{z}{\left(\left(o^{{\left(\psi_{w_{i}}\right)}^{t}}-1\right)\right)}^{R_{i}}\right)} \end{array}\right)$$

$$=\left(\left(\begin{array}{c} \sqrt[{t}]{1-\log_{o}\left(1+\left(o^{1-v_{i}^{t}}-1\right)\right)},\\ e^{i2\pi \sqrt[{t}]{1-\log_{o}\left(1+\left(o^{1-{\left(\theta_{v_{i}}\right)}^{t}}-1\right)\right)}} \end{array}\right),\left(\begin{array}{c} \log_{o}\left(1+\left(o^{u_{i}^{t}}-1\right)\right),\\ e^{i2\pi \;\log_{o}\left(1+\left(o^{{\left(\varphi_{u_{i}}\right)}^{t}}-1\right)\right)} \end{array}\right),\left(\left(\begin{array}{c} \log_{o}\left(1+\left(o^{w_{i}^{t}}-1\right)\right),\\ e^{i2\pi \;\log_{o}\left(1+\left(o^{{\left(\psi_{w_{i}}\right)}^{t}}-1\right)\right)} \end{array}\right)\right)\right)$$

$$=\left(\left(v,e^{i2\pi \theta_{v}}\right),\left(u,e^{i2\pi \varphi_{u}}\right),\left(w,e^{i2\pi \psi_{w}}\right)\right)=q$$

**Proof of**Theorem 4**:** Suppose $q_{i}=\left(v_{i}.\;e^{i2\pi \theta_{v_{i}}},u_{i}.\;e^{i2\pi \varphi_{u_{i}}},w_{i}.\;e^{i2\pi \psi_{w_{i}}}\right)$ be an aggregation of CTSFFVs. Suppose $q_{i}^{-}=\min\left(q_{1},q_{2},\ldots ,q_{n}\right)=\left(v_{i}^{-}.e^{i2\pi \theta_{v_{i}}^{-}},u_{i}^{-}.e^{i2\pi \varphi_{u_{i}}^{-}},w_{i}^{-}.e^{i2\pi \psi_{w_{i}}^{-}}\right)$ and $q_{i}^{+}=\min\left(q_{1},q_{2},\ldots ,q_{n}\right)=\left(v_{i}^{+}.e^{i2\pi \theta_{v_{q}}^{+}},u_{i}^{+}.e^{i2\pi \varphi_{u_{i}}^{+}},w_{i}^{+}.e^{i2\pi \psi_{w_{i}}^{+}}\right)$. We find $v_{i}^{-}=\min\left(v_{i}\right)$, $e^{i2\pi \theta_{v_{i}}^{-}}=\min\left(\;e^{i2\pi \theta_{v_{i}}}\right)$, $u_{i}^{-}=\max\left(u_{i}\right)$, $e^{i2\pi \varphi_{u_{i}}^{-}}=\max\left(e^{i2\pi \varphi_{u_{i}}}\right)$, $w_{i}^{-}=\max\left(w_{i}\right)$ and $e^{i2\pi \psi_{w_{i}}^{-}}=\max\left(\;e^{i2\pi \psi_{w_{i}}}\right)$, $v_{i}^{-}=\max\left(v_{i}\right)$, $e^{i2\pi \theta_{v_{i}}^{+}}=\max\left(e^{i2\pi \theta_{v_{i}}}\right)$, $u_{i}^{-}=\min\left(u_{i}\right)$, $e^{i2\pi \varphi_{u_{i}}^{+}}=\min\left(\;e^{i2\pi \varphi_{u_{i}}}\right)$, $w_{i}^{-}=\min\left(w_{i}\right)$ and $e^{i2\pi \psi_{w_{i}}^{+}}=\min\left(e^{i2\pi \psi_{w_{i}}}\right)$. So, we can find inequality:

$$=\left(\begin{array}{c} \sqrt[{t}]{1-\log_{o}\left(1+\prod_{i=1}^{n}{\left(\left(o^{1-{v^{-}}_{i}^{t}}-1\right)\;\right)}^{R_{i}}\;\right)},\\ e^{i2\pi \sqrt[{t}]{1-\log_{o}\left(1+\prod_{i=1}^{n}{\left(\left(o^{1-{\left(\theta_{v_{i}}^{-}\right)}^{t}}-1\right)\;\right)}^{R_{i}}\right)}} \end{array}\right)\leq \left(\begin{array}{c} \sqrt[{t}]{1-\log_{o}\left(1+\prod_{i=1}^{n}{\left(\left(o^{1-v_{i}^{t}}-1\right)\;\right)}^{R_{i}}\;\right)},\\ e^{i2\pi \sqrt[{t}]{1-\log_{o}\left(1+\prod_{i=1}^{n}{\left(\left(o^{1-{\left(\theta_{v_{i}}\right)}^{t}}-1\right)\;\right)}^{R_{i}}\right)}} \end{array}\right)\leq \left(\begin{array}{c} \sqrt[{t}]{1-\log_{o}\left(1+\prod_{i=1}^{n}{\left(\left(o^{1-{v^{+}}_{i}^{t}}-1\right)\;\right)}^{R_{i}}\;\right)},\\ e^{i2\pi \sqrt[{t}]{1-\log_{o}\left(1+\prod_{i=1}^{n}{\left(\left(o^{1-{\left(\theta_{v_{i}}^{+}\right)}^{t}}-1\right)\;\right)}^{R_{i}}\right)}} \end{array}\right)$$

$$=\left(\begin{array}{c} \log_{o}\left(1+\prod_{i=1}^{n}\left({\left(o^{{u^{-}}_{i}^{t}}-1\right)}^{R_{i}}\right)\right),\\ e^{i2\pi \;\log_{o}\left(1+\prod_{i=1}^{n}\left({\left(o^{{\left(\varphi_{u_{i}}^{-}\right)}^{t}}-1\right)}^{R_{i}}\right)\right)} \end{array}\right)\leq \left(\begin{array}{c} \log_{o}\left(1+\prod_{i=1}^{n}\left({\left(o^{u_{i}^{t}}-1\right)}^{R_{i}}\right)\right),\\ e^{i2\pi \;\log_{o}\left(1+\prod_{i=1}^{n}\left({\left(o^{{\left(\varphi_{u_{i}}\right)}^{t}}-1\right)}^{R_{i}}\right)\right)} \end{array}\right)\leq \left(\begin{array}{c} \log_{o}\left(1+\prod_{i=1}^{n}\left({\left(o^{{u^{+}}_{i}^{t}}-1\right)}^{R_{i}}\right)\right),\\ e^{i2\pi \;\log_{o}\left(1+\prod_{i=1}^{n}\left({\left(o^{\left(\varphi_{u_{i}}^{+}\right)}-1\right)}^{R_{i}}\right)\right)} \end{array}\right)$$

$$=\left(\begin{array}{c} \log_{o}\left(1+\prod_{i=1}^{n}\left({\left(o^{{w^{-}}_{i}^{t}}-1\right)}^{R_{i}}\right)\right),\\ e^{i2\pi \;\log_{o}\left(1+\prod_{i=1}^{n}\left({\left(o^{{\left(\psi_{w_{i}}^{-}\right)}^{t}}-1\right)}^{R_{i}}\right)\right)} \end{array}\right)\leq \left(\begin{array}{c} \log_{o}\left(1+\prod_{i=1}^{n}\left({\left(o^{w_{i}^{t}}-1\right)}^{R_{i}}\right)\right),\\ e^{i2\pi \;\log_{o}\left(1+\prod_{i=1}^{n}\left({\left(o^{{\left(\psi_{w_{i}}\right)}^{t}}-1\right)}^{R_{i}}\right)\right)} \end{array}\right)\leq \left(\begin{array}{c} \log_{o}\left(1+\prod_{i=1}^{n}\left({\left(o^{{w^{+}}_{i}^{t}}-1\right)}^{R_{i}}\right)\right),\\ e^{i2\pi \;\log_{o}\left(1+\prod_{i=1}^{n}\left({\left(o^{{\left(\psi_{w_{i}}^{+}\right)}^{t}}-1\right)}^{R_{i}}\right)\right)} \end{array}\right)$$

So,

$$q_{i}^{-}\leq CTSFFWA\left(q_{1},q_{2},\ldots ,q_{n}\right)\leq q_{i}^{+}$$

**Proof of**Theorem 5**:** Let two CTSFFVs $q_{i}=\left(v_{i}.\;e^{i2\pi \theta_{v_{i}}},u_{i}.\;e^{i2\pi \varphi_{u_{i}}},w_{i}.\;e^{i2\pi \psi_{w_{i}}}\right)$ and $\overset{´}{q_{i}}=\left(\overset{´}{v_{i}}.\;e^{i2\pi \overset{´}{\theta_{v_{i}}}},\overset{´}{u_{i}}.\;e^{i2\pi \overset{´}{\varphi_{u_{i}}}},\overset{´}{w_{i}}.\;e^{i2\pi \overset{´}{\psi_{w_{i}}}}\right)$ and i=(1,2,…,n). In this value, **we** can apply $q_{i}\leq \overset{´}{q_{i}}$.

$$\left(\begin{array}{c} \sqrt[{t}]{1-\log_{o}\left(1+\prod_{i=1}^{n}{\left(\left(o^{1-v_{i}^{t}}-1\right)\;\right)}^{R_{i}}\;\right)},\\ e^{i2\pi \sqrt[{t}]{1-\log_{o}\left(1+\prod_{i=1}^{n}{\left(\left(o^{1-{\left(\theta_{v_{i}}\right)}^{t}}-1\right)\;\right)}^{R_{i}}\right)}} \end{array}\right)\leq \left(\begin{array}{c} \sqrt[{t}]{1-\log_{o}\left(1+\prod_{i=1}^{n}{\left(\left(o^{1-{\overset{´}{v}}_{i}^{t}}-1\right)\;\right)}^{R_{i}}\;\right)},\\ e^{i2\pi \sqrt[{t}]{1-\log_{o}\left(1+\prod_{i=1}^{n}{\left(\left(o^{1-{\left(\overset{´}{\theta_{v_{i}}}\right)}^{t}}-1\right)\;\right)}^{R_{i}}\right)}} \end{array}\right)$$

$$\left(\begin{array}{c} \log_{o}\left(1+\prod_{i=1}^{n}\left({\left(o^{u_{i}^{t}}-1\right)}^{R_{i}}\right)\right),\\ e^{i2\pi \;\log_{o}\left(1+\prod_{i=1}^{n}\left({\left(o^{{\left(\varphi_{u_{i}}\right)}^{t}}-1\right)}^{R_{i}}\right)\right)} \end{array}\right)\leq \left(\begin{array}{c} \log_{o}\left(1+\prod_{i=1}^{n}\left({\left(o^{{\overset{´}{u}}_{i}^{t}}-1\right)}^{R_{i}}\right)\right),\\ e^{i2\pi \;\log_{o}\left(1+\prod_{i=1}^{n}\left({\left(o^{{\left(\overset{´}{\varphi_{u_{i}}}\right)}^{t}}-1\right)}^{R_{i}}\right)\right)} \end{array}\right)$$

$$\left(\begin{array}{c} \log_{o}\left(1+\prod_{i=1}^{n}\left({\left(o^{w_{i}^{t}}-1\right)}^{R_{i}}\right)\right),\\ e^{i2\pi \;\log_{o}\left(1+\prod_{i=1}^{n}\left({\left(o^{{\left(\psi_{w_{i}}\right)}^{t}}-1\right)}^{R_{i}}\right)\right)} \end{array}\right)\leq \left(\begin{array}{c} \log_{o}\left(1+\prod_{i=1}^{n}\left({\left(o^{{\overset{´}{w}}_{i}^{t}}-1\right)}^{R_{i}}\right)\right),\\ e^{i2\pi \;\log_{o}\left(1+\prod_{i=1}^{n}\left({\left(o^{{\left(\overset{´}{\psi_{w_{i}}}\right)}^{t}}-1\right)}^{R_{i}}\right)\right)} \end{array}\right)$$

So,

$$CTSFFPWA\left(q_{1},q_{2},\ldots ,q_{n}\right)\leq CTSFFPWA\left({\overset{´}{q}}_{1},{\overset{´}{q}}_{2},\ldots ,\overset{´}{q_{n}}\right)$$

**Proof of**Theorem 8**:** We can use induction of the mathematics rule:

$$q_{1}R_{1}=\left(\begin{array}{c} \log_{o}\left(1+{\left(o^{1-v_{1}^{t}}-1\right)}^{R_{1}}\right),e^{i2\pi \left(\log_{o}\left(1+{\left(o^{1-{\left(\theta_{v_{1}}\right)}^{t}}-1\right)}^{R_{1}}\right)\right)}\\ \sqrt[{t}]{1-\log_{o}\left(1+{\left(o^{u_{1}^{t}}-1\right)}^{R_{1}}\right)},e^{i2\pi \sqrt[{t}]{1-\log_{o}\left(1+{\left(o^{{\left(\varphi_{u_{1}}\right)}^{t}}-1\right)}^{R_{1}}\right)}}\\ \sqrt[{t}]{1-\log_{o}\left(1+{\left(o^{w_{1}^{t}}-1\right)}^{R_{1}}\right)},e^{i2\pi \sqrt[{t}]{1-\log_{o}\left(1+{\left(o^{{\left(\psi_{w_{1}}\right)}^{t}}-1\right)}^{R_{1}}\right)}} \end{array}\right)$$

$$q_{2}R_{2}=\left(\begin{array}{c} \log_{o}\left(1+{\left(o^{1-v_{2}^{t}}-1\right)}^{R_{2}}\right),e^{i2\pi \left(\log_{o}\left(1+{\left(o^{1-{\left(\theta_{v_{2}}\right)}^{t}}-1\right)}^{R_{2}}\right)\right)}\\ \sqrt[{t}]{1-\log_{o}\left(1+{\left(o^{u_{2}^{t}}-1\right)}^{R_{2}}\right)},e^{i2\pi \sqrt[{t}]{1-\log_{o}\left(1+{\left(o^{{\left(\varphi_{u_{2}}\right)}^{t}}-1\right)}^{R_{2}}\right)}}\\ \sqrt[{t}]{1-\log_{o}\left(1+{\left(o^{w_{2}^{t}}-1\right)}^{R_{2}}\right)},e^{i2\pi \sqrt[{t}]{1-\log_{o}\left(1+{\left(o^{{\left(\psi_{w_{2}}\right)}^{t}}-1\right)}^{R_{2}}\right)}} \end{array}\right)$$

By using the **definition**:

$$CTSFFPWA\left(q_{1},q_{2}\right)=q_{1}R_{1}⊕q_{2}R_{2}=\left(\begin{array}{c} \log_{o}\left(1+{\left(o^{1-v_{1}^{t}}-1\right)}^{R_{1}}\right),e^{i2\pi \left(\log_{o}\left(1+{\left(o^{1-{\left(\theta_{v_{1}}\right)}^{t}}-1\right)}^{R_{1}}\right)\right)}\\ \sqrt[{t}]{1-\log_{o}\left(1+{\left(o^{u_{1}^{t}}-1\right)}^{R_{1}}\right)},e^{i2\pi \sqrt[{t}]{1-\log_{o}\left(1+{\left(o^{{\left(\varphi_{u_{1}}\right)}^{t}}-1\right)}^{R_{1}}\right)}}\\ \sqrt[{t}]{1-\log_{o}\left(1+{\left(o^{w_{1}^{t}}-1\right)}^{R_{1}}\right)},e^{i2\pi \sqrt[{t}]{1-\log_{o}\left(1+{\left(o^{{\left(\psi_{w_{1}}\right)}^{t}}-1\right)}^{R_{1}}\right)}} \end{array}\right)⊕\left(\begin{array}{c} \log_{o}\left(1+{\left(o^{1-v_{2}^{t}}-1\right)}^{R_{2}}\right),e^{i2\pi \left(\log_{o}\left(1+{\left(o^{1-{\left(\theta_{v_{2}}\right)}^{t}}-1\right)}^{R_{2}}\right)\right)}\\ \sqrt[{t}]{1-\log_{o}\left(1+{\left(o^{u_{2}^{t}}-1\right)}^{R_{2}}\right)},e^{i2\pi \sqrt[{t}]{1-\log_{o}\left(1+{\left(o^{{\left(\varphi_{u_{2}}\right)}^{t}}-1\right)}^{R_{2}}\right)}}\\ \sqrt[{t}]{1-\log_{o}\left(1+{\left(o^{w_{2}^{t}}-1\right)}^{R_{2}}\right)},e^{i2\pi \sqrt[{t}]{1-\log_{o}\left(1+{\left(o^{{\left(\psi_{w_{2}}\right)}^{t}}-1\right)}^{R_{2}}\right)}} \end{array}\right)$$

$$=\left(\begin{array}{c} \log_{o}\left(1+{\left(o^{1-v_{1}^{t}}-1\right)}^{R_{1}}{\left(o^{1-v_{2}^{t}}-1\right)}^{R_{2}}\right),e^{i2\pi \left(\log_{o}\left(1+{\left(o^{1-{\left(\theta_{v_{1}}\right)}^{t}}-1\right)}^{R_{1}}{\left(o^{1-{\left(\theta_{v_{2}}\right)}^{t}}-1\right)}^{R_{2}}\right)\right)}\\ \sqrt[{t}]{1-\log_{o}\left(1+{\left(o^{u_{1}^{t}}-1\right)}^{R_{1}}{\left(o^{u_{2}^{t}}-1\right)}^{R_{2}}\right)},e^{i2\pi \sqrt[{t}]{1-\log_{o}\left(1+{\left(o^{\varphi_{1}^{t}}-1\right)}^{R_{1}}{\left(o^{\varphi_{2}^{t}}-1\right)}^{R_{2}}\right)}}\\ \sqrt[{t}]{1-\log_{o}\left(1+{\left(o^{w_{1}^{t}}-1\right)}^{R_{1}}{\left(o^{w_{2}^{t}}-1\right)}^{R_{2}}\right)},e^{i2\pi \sqrt[{t}]{1-\log_{o}\left(1+{\left(o^{\psi_{1}^{t}}-1\right)}^{R_{1}}{\left(o^{\psi_{2}^{t}}-1\right)}^{R_{2}}\right)}} \end{array}\right)$$

$$=\left(\begin{array}{c} \log_{o}\left(1+\prod_{i=1}^{2}{\left(\left(o^{1-v_{i}^{t}}-1\right)\;\right)}^{R_{i}}\;\right),e^{i2\pi \left(\log_{o}\left(1+\prod_{i=1}^{2}{\left(\left(o^{1-{\left(\theta_{v_{i}}\right)}^{t}}-1\right)\;\right)}^{R_{i}}\right)\right)}\\ \sqrt[{t}]{1-\log_{o}\left(1+\prod_{i=1}^{2}\left({\left(o^{u_{i}^{t}}-1\right)}^{R_{i}}\right)\right)},e^{i2\pi \sqrt[{t}]{1-\log_{o}\left(1+\prod_{i=1}^{2}\left({\left(o^{{\left(\varphi_{u_{i}}\right)}^{t}}-1\right)}^{R_{i}}\right)\right)}}\\ \sqrt[{t}]{1-\log_{o}\left(1+\prod_{i=1}^{2}\left({\left(o^{w_{i}^{t}}-1\right)}^{R_{i}}\right)\right)},e^{i2\pi \sqrt[{t}]{1-\log_{o}\left(1+\prod_{i=1}^{2}\left({\left(o^{{\left(\psi_{w_{i}}\right)}^{t}}-1\right)}^{R_{i}}\right)\right)}} \end{array}\right)$$

For n=k.

$$=\left(\begin{array}{c} \log_{o}\left(1+\prod_{i=1}^{k}{\left(\left(o^{1-v_{i}^{t}}-1\right)\;\right)}^{R_{i}}\;\right),e^{i2\pi \left(\log_{o}\left(1+\prod_{i=1}^{k}{\left(\left(o^{1-{\left(\theta_{v_{i}}\right)}^{t}}-1\right)\;\right)}^{R_{i}}\right)\right)}\\ \sqrt[{t}]{1-\log_{o}\left(1+\prod_{i=1}^{k}\left({\left(o^{u_{i}^{t}}-1\right)}^{R_{i}}\right)\right)},e^{i2\pi \sqrt[{t}]{1-\log_{o}\left(1+\prod_{i=1}^{k}\left({\left(o^{{\left(\varphi_{u_{i}}\right)}^{t}}-1\right)}^{R_{i}}\right)\right)}}\\ \sqrt[{t}]{1-\log_{o}\left(1+\prod_{i=1}^{k}\left({\left(o^{w_{i}^{t}}-1\right)}^{R_{i}}\right)\right)},e^{i2\pi \sqrt[{t}]{1-\log_{o}\left(1+\prod_{i=1}^{k}\left({\left(o^{{\left(\psi_{w_{i}}\right)}^{t}}-1\right)}^{R_{i}}\right)\right)}} \end{array}\right)$$

For n=k+1.

$$=\left(\begin{array}{c} \log_{o}\left(1+\prod_{i=1}^{k}{\left(\left(o^{1-v_{i}^{t}}-1\right)\;\right)}^{R_{i}}\;\right),e^{i2\pi \left(\log_{o}\left(1+\prod_{i=1}^{k}{\left(\left(o^{1-{\left(\theta_{v_{i}}\right)}^{t}}-1\right)\;\right)}^{R_{i}}\right)\right)}\\ \sqrt[{t}]{1-\log_{o}\left(1+\prod_{i=1}^{k}\left({\left(o^{u_{i}^{t}}-1\right)}^{R_{i}}\right)\right)},e^{i2\pi \sqrt[{t}]{1-\log_{o}\left(1+\prod_{i=1}^{k}\left({\left(o^{{\left(\varphi_{u_{i}}\right)}^{t}}-1\right)}^{R_{i}}\right)\right)}}\\ \sqrt[{t}]{1-\log_{o}\left(1+\prod_{i=1}^{k}\left({\left(o^{w_{i}^{t}}-1\right)}^{R_{i}}\right)\right)},e^{i2\pi \sqrt[{t}]{1-\log_{o}\left(1+\prod_{i=1}^{k}\left({\left(o^{{\left(\psi_{w_{i}}\right)}^{t}}-1\right)}^{R_{i}}\right)\right)}} \end{array}\right)⊕\left(\begin{array}{c} \log_{o}\left(1+\prod_{i=1}^{k+1}{\left(\left(o^{1-v_{i}^{t}}-1\right)\;\right)}^{R_{i}}\;\right),e^{i2\pi \left(\log_{o}\left(1+\prod_{i=1}^{k+1}{\left(\left(o^{1-{\left(\theta_{v_{i}}\right)}^{t}}-1\right)\;\right)}^{R_{i}}\right)\right)}\\ \sqrt[{t}]{1-\log_{o}\left(1+\prod_{i=1}^{k+1}\left({\left(o^{u_{i}^{t}}-1\right)}^{R_{i}}\right)\right)},e^{i2\pi \sqrt[{t}]{1-\log_{o}\left(1+\prod_{i=1}^{k+1}\left({\left(o^{{\left(\varphi_{u_{i}}\right)}^{t}}-1\right)}^{R_{i}}\right)\right)}}\\ \sqrt[{t}]{1-\log_{o}\left(1+\prod_{i=1}^{k+1}\left({\left(o^{w_{i}^{t}}-1\right)}^{R_{i}}\right)\right)},e^{i2\pi \sqrt[{t}]{1-\log_{o}\left(1+\prod_{i=1}^{k+1}\left({\left(o^{{\left(\psi_{w_{i}}\right)}^{t}}-1\right)}^{R_{i}}\right)\right)}} \end{array}\right)$$

$$=\left(\begin{array}{c} \log_{o}\left(1+\prod_{i=1}^{k+1}{\left(\left(o^{1-v_{i}^{t}}-1\right)\;\right)}^{R_{i}}\;\right),e^{i2\pi \left(\log_{o}\left(1+\prod_{i=1}^{k+1}{\left(\left(o^{1-{\left(\theta_{v_{i}}\right)}^{t}}-1\right)\;\right)}^{R_{i}}\right)\right)}\\ \sqrt[{t}]{1-\log_{o}\left(1+\prod_{i=1}^{k+1}\left({\left(o^{u_{i}^{t}}-1\right)}^{R_{i}}\right)\right)},e^{i2\pi \sqrt[{t}]{1-\log_{o}\left(1+\prod_{i=1}^{k+1}\left({\left(o^{{\left(\varphi_{u_{i}}\right)}^{t}}-1\right)}^{R_{i}}\right)\right)}}\\ \sqrt[{t}]{1-\log_{o}\left(1+\prod_{i=1}^{k+1}\left({\left(o^{w_{i}^{t}}-1\right)}^{R_{i}}\right)\right)},e^{i2\pi \sqrt[{t}]{1-\log_{o}\left(1+\prod_{i=1}^{k+1}\left({\left(o^{{\left(\psi_{w_{i}}\right)}^{t}}-1\right)}^{R_{i}}\right)\right)}} \end{array}\right)$$

So, this expression **is** true for n=k+1.
